# Converged Structural and Spectroscopic Properties for Refined QM/MM Models of Azurin

**DOI:** 10.1021/acs.inorgchem.1c00640

**Published:** 2021-05-03

**Authors:** Christine E. Schulz, Maurice van Gastel, Dimitrios A. Pantazis, Frank Neese

**Affiliations:** Max-Planck-Institut für Kohlenforschung, Kaiser-Wilhelm-Platz 1, 45470 Mülheim an der Ruhr, Germany

## Abstract

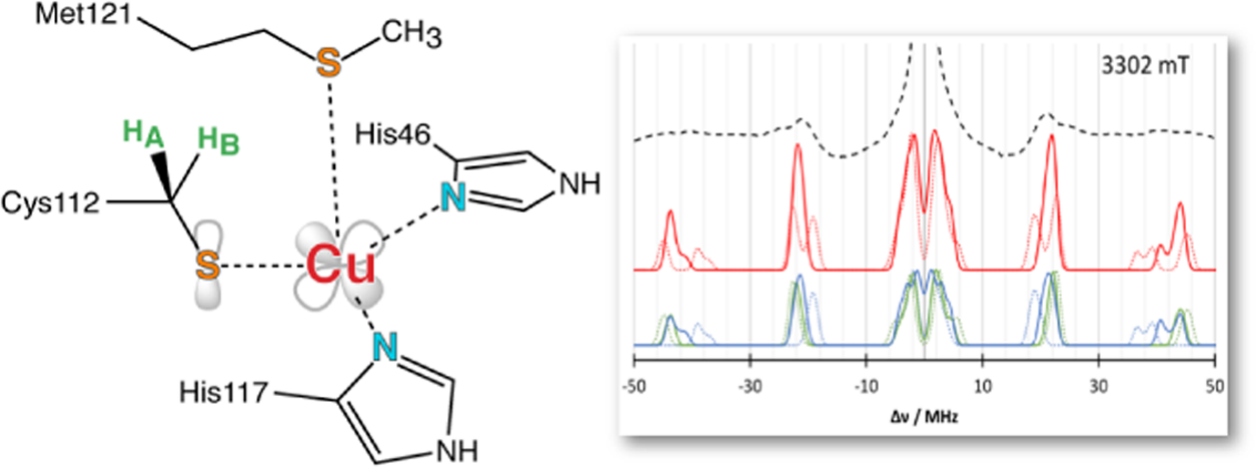

Blue copper proteins continue to challenge experiment and theory with their electronic structure and spectroscopic properties that respond sensitively to the coordination environment of the copper ion. In this work, we report state-of-the art electronic structure studies for geometric and spectroscopic properties of the archetypal “Type I” copper protein azurin in its Cu(II) state. A hybrid quantum mechanics/molecular mechanics (QM/MM) approach is used, employing both density functional theory (DFT) and coupled cluster with singles, doubles, and perturbative triples (CCSD(T)) methods for the QM region, the latter method making use of the domain-based local pair natural orbital (DLPNO) approach. Models of increasing QM size are employed to investigate the convergence of critical geometric parameters. It is shown that convergence is slow and that a large QM region is critical for reproducing the short experimental Cu–SCys112 distance. The study of structural convergence is followed by investigation of spectroscopic parameters using both DFT and DLPNO-CC methods and comparing these to the experimental spectrum using simulations. The results allow us to examine for the first time the distribution of spin densities and hyperfine coupling constants at the coupled cluster level, leading us to revisit the experimental assignment of the ^33^S hyperfine splitting. The wavefunction-based approach to obtain spin-dependent properties of open-shell systems demonstrated here for the case of azurin is transferable and applicable to a large array of bioinorganic systems.

## Abstract 2

The electronic structure and the structure−spectroscopy correlations for the Cu(II) center of azurin are revisited with calculations of spin densities and hyperfine coupling constants at the coupled cluster level of theory.

## 1 Introduction

Copper is the second most abundant transition metal in biological systems,^1^ and Cu-containing enzymes are known to catalyze a variety of reactions, in addition to being involved in electron transfer processes. The copper centers can be categorized based on geometry and coordination.^2,3^ Type I Cu centers, also called blue copper centers, feature an intense absorption at around 600 nm. Type II or “normal” low-molecular-weight copper coordination compounds lack this absorption.^4^ Besides these, dimeric type III copper proteins and artificial classes (e.g., “type-0” copper^5^) exist.^6^ Aside from their UV/vis spectra, these types can be distinguished by electron paramagnetic resonance (EPR) spectroscopy.^7^ Because of the unusual active-site geometry compared to synthetic, tetrahedral, or square planar Cu(II) complexes, the blue copper proteins gave rise to the question if the protein is following the active site, or if the active site geometry is dictated by the protein matrix (entatic state principle).^8−11^

**Figure 1:**
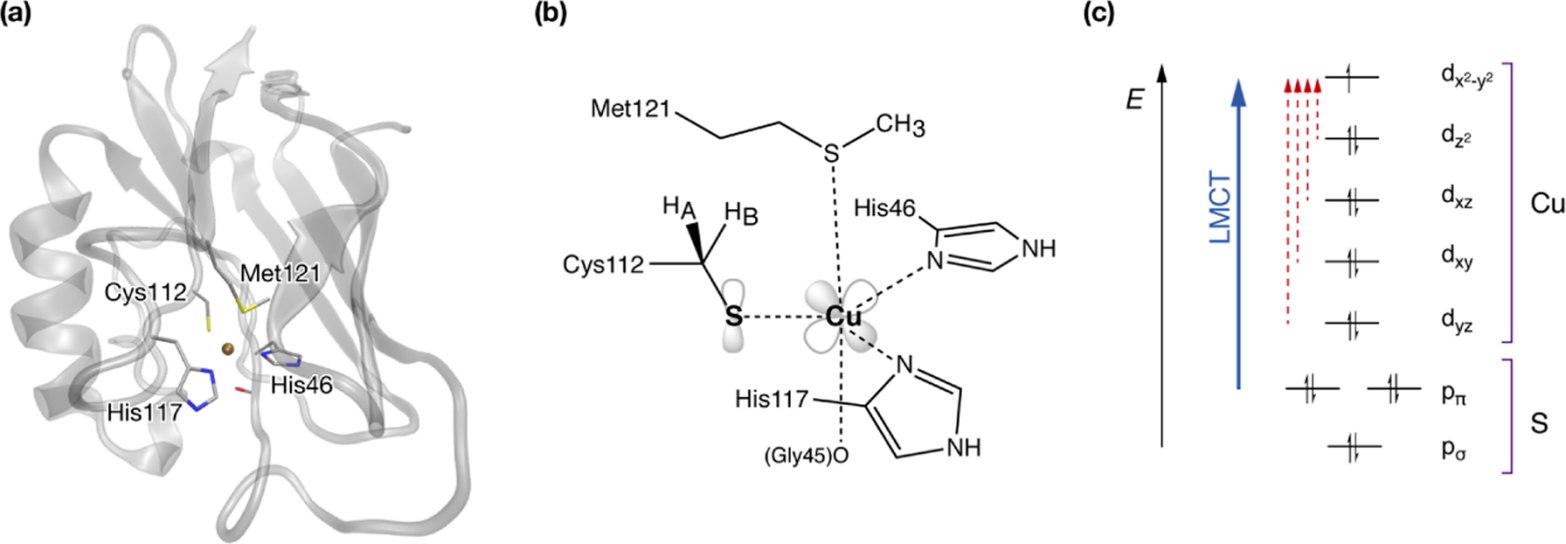
Structure of azurin (a), schematic depiction of the copper site including the SOMO (b), and molecular orbital picture (c) showing the name-giving LMCT absorption.

Azurin is a representative type I copper protein with a distorted trigonal bipyramidal active-site geometry.^12^ It mediates one-electron transfer in bacteria by switching the oxidation state of copper between Cu(I) and Cu(II). The copper ion is ligated by a cysteinate (Cys112) and two histidine residues (His46 and His117) in the ligand plane, as well as a weakly bound methionine (Met121) and the backbone carbonyl of Gly45 as axial ligands (Figure 1). This active site geometry, specifically the thiolate sulfur coordination, gives rise to unique spectral features,^13^ which are known to be modulated by the covalency of the Cu–S_Cys_ bond. The origin of the bright absorption in the Cu(II) state has been clarified in pioneering in-depth studies by Solomon and co-workers and is attributed to ligand-to-metal charge transfer (LMCT) involving the cysteine p_π_ and the half-occupied copper d_*x*^2^–*y*^2^_-based molecular orbitals.^14,15^ This is contrary to molecular, normal, copper complexes, often represented by the square planar CuCl_4_.^16^ Their absorption spectrum is dominated by transitions from the ligand p_σ_ orbitals to copper d_*x*^2^–*y*^2^_, while the π transition is of smaller intensity—an inverted intensity pattern compared to blue copper proteins.^17^

The strong LMCT bands in azurin indicate a strongly covalent bond between copper and cysteine sulfur. The quantification of the covalency at the copper center has been the subject of various studies utilizing a wide range of spectroscopic techniques. One method is sulfur K-edge X-ray absorption spectroscopy (XAS), where the intensity of the pre-edge region is determined by the 3p/3d mixing.^18,19^ Another approach that gives a broader picture of the coordination environment of copper is EPR spectroscopy and associated techniques. The hyperfine coupling constants (HFCs) obtained from these experiments report on the interaction of the nuclear spin of a specific isotope with the electron spin of the system. In addition to copper,^20^ the histidine nitrogens N_δ_ and N_ε_, and hydrogen HFCs have been measured.^21,22^ Several studies also focused on the HFCs of the cysteine β-hydrogens (H_A_, H_B_) to elucidate the distribution of spin density on the cysteine ligand.^22−25^ Overall, diverse interpretations of experimental data are encountered in different studies.

Using XAS Cu K-edge spectroscopy,^18,19^ the pre-edge intensity indicates how much *s* character is mixed into the Cu 3d_*x*^2^–*y*^2^_ orbital. To extract the covalency of the ligand–metal bond, in the sense of the amount of ligand character, the pre-edge area is fitted and compared to a reference.^26^ In wild-type azurin, the singly occupied molecular orbital (SOMO) was determined to have 40 ± 3% sulfur character.^18^ Since there are two sulfur-containing ligands to the copper (Cys112 and Met121), the experiment was redone using selenomethionine (M121SeM) mutants, showing a sulfur character of 37.5 ± 3%, which is arising only from the Cys112 sulfur. Hence, the covalency of the Cu–SCys112 bond was characterized as 37.5% covalency at the sulfur. Using point mutations, also the second coordination sphere was investigated.^19^ In that study, in wild-type azurin, a sulfur character of 45 ± 3% was determined. Removing the hydrogen bond to cysteine sulfur in an F114P mutant leads to an increase of the sulfur character to 54 ± 3%. Further changes in the second solvation sphere, which have been attributed to changes in the electrostatic environment of the copper center, lead to a decrease in the sulfur character, to 31 ± 3% for N47S and 43 ± 3% for F114N azurin, respectively.

In contrast to the isolated fitting process involved in the determination of covalency using XAS, EPR spectroscopy requires fitting of spin Hamiltonian parameters for the whole copper ligand system. This leads to a steep increase in complexity. Therefore, in the latest electron–electron double resonance detected nuclear magnetic resonance (EDNMR) study,^27^ the results from quantum mechanics/molecular mechanics (QM/MM) calculations,^28^ one-dimensional (1D) EDNMR, and two-dimensional (2D) EDNMR are compared. Unfortunately, this leads to different sets of spin Hamiltonian parameters, with the sulfur HFC differing by 10 MHz. Also, to improve the fit, the principal values of the ^14^N HFCs have been varied, leading to an increase of 2 MHz with respect to previous experiments.^21^ To extract the spin population from the ^33^S HFCs empirical comparisons were used,^29^ which yield a total sulfur spin population of 29.1–30.4%, a value that suggests significantly lower covalency of the Cu–S bond compared to 38% inferred from XAS.^18,19^ It is also much lower than the spin population determined by previous spectroscopy-connected computational studies (36–62%).^28,30−32^

To rationalize these differences in interpretation, the concepts involved in the transfer from the experiment to a descriptor of the electronic structure, such as the spin population are reviewed. There are three main conceptual points here: (1) The experimental procedure and post-treatment, especially in the case of the HFCs for the fitting procedure of the spin Hamiltonian (SH) parameters; (2) extraction of the spin population from the experiment, in the case of the HFCs from the SH parameters; and (3) the concept of spin population and its generality. As pointed out previously,^33,34^ spin populations are not physical observables, and hence there is not necessarily a uniquely best definition. The spin density distribution is a three-dimensional (3D) function of space and is an observable property from which other observables like hyperfine couplings can be unambiguously deduced. The spin population is a more or less arbitrary assignment of spin density to individual atoms in the system according to some prescription. It also takes no notice of the actual radial shape of the spin density distribution and rather represents an integrated quantity. Consequently, the relationship between spin population and physical observables involves approximations that are different for each spectroscopic method. Hence, it is not surprising when the values for spin populations deduced from different experimental methods do not match. Nevertheless, we use spin populations for qualitative interpretative purposes. In particular, in the case of the type 1 copper site, values of the sulfur spin population deduced from density functional theory (DFT) calculations tend to be much higher (50%) than values deduced either from XAS (38%)^19^ or EPR/ENDOR (30%).^27^ This is a significant discrepancy, on which we hope to shed more light in the present study that uses state-of-the-art quantum chemical modeling of the type 1 copper site in Azurin.

The first step in obtaining an accurate picture of the electronic structure at the copper center is to describe the underlying geometric structure as accurately as possible. The Cu–S_Cys_ distance is an important structural parameter that has been ill-defined in crystallographic studies, which historically present a wide distribution of values from 2.20 to 2.30 Å. Extended X-ray absorption fine structure (EXAFS) studies^35^ suggested a shorter Cu–S distance and the most recent EXAFS study indicates a length of 2.12 Å for the Cu–S bond,^36^ shorter than most crystallographic models. Computational studies have addressed many of the structural and spectroscopic properties of copper proteins using a variety of theoretical approaches, ranging from semiempirical^14,37,38^ to multireference methods^10,39,40^ and excited-state dynamics.^41−43^ The protein has been sometimes treated using molecular mechanics^44,45^ or QM/MM.^28,46−48^ Spectroscopic properties have been calculated using density functional theory (DFT). One example is the azurin *g*-tensor and hyperfine couplings.^32^ In a QM/MM framework, optical and X-ray absorption spectra as well as hyperfine coupling constants have been determined.^28^ However, methods more accurate and reliable than DFT were not generally feasible for realistic models of azurin that include the protein environment.

Here we revisit the geometric and electronic structure and properties of azurin in the Cu(II) oxidation state using a QM/MM approach to include an explicit description of the protein environment. The complete solvated protein is included in the MM region. To ensure the best possible geometries are obtained, the performance of different DFT functionals is first benchmarked against coupled cluster theory for structural parameters. This is possible thanks to the domain-based local pair natural orbital implementation of coupled cluster singles doubles and perturbative triples, DLPNO-CCSD(T),^49^ that is available for open-shell systems.^50^ The convergence of spin densities is carefully studied and ligand HFCs are computed by both DFT and DLPNO-CCSD methods.^51^ The results derived from a sequence of QM/MM models describe how the spin density distribution responds to specific components of the protein, while the spectroscopic parameters are analyzed in detail and compared with experimental data to resolve the conflicting interpretations of experimental studies regarding the Cu–S covalency and provide insight into the structure–property correlations for blue copper sites.

## 2 Models and Methods

### 2.1 Construction of the QM/MM Model

The MM models are based on an average geometry of the four tetramers in the 4AZU^52^ structure.^53^ Starting from the crystal structure, protons were added for pH 7 using the CHARMM36 force-field parameters.^54^ Standard protonation states were assumed for all residues. The histidines were assumed to be protonated in the N_ε_ position, unless indicated otherwise by nearby residues. TIP3P water molecules were added to the protein, forming a sphere with at least 5 Å of buffering between the protein surface and the surface of the sphere. This adds 1459 water molecules in total. Hydrogen positions were minimized with a 10 000-step conjugate gradient algorithm. Water positions were relaxed using a 10 000-step NVT simulation at 100 K, while the oxygens of the outer water layer were kept fixed at their initial positions to prevent “breathing” motions of the system. Finally, both hydrogen and water positions were optimized using a 10 000-step conjugate gradient algorithm, with the same constraints employed.

**Figure 2:**
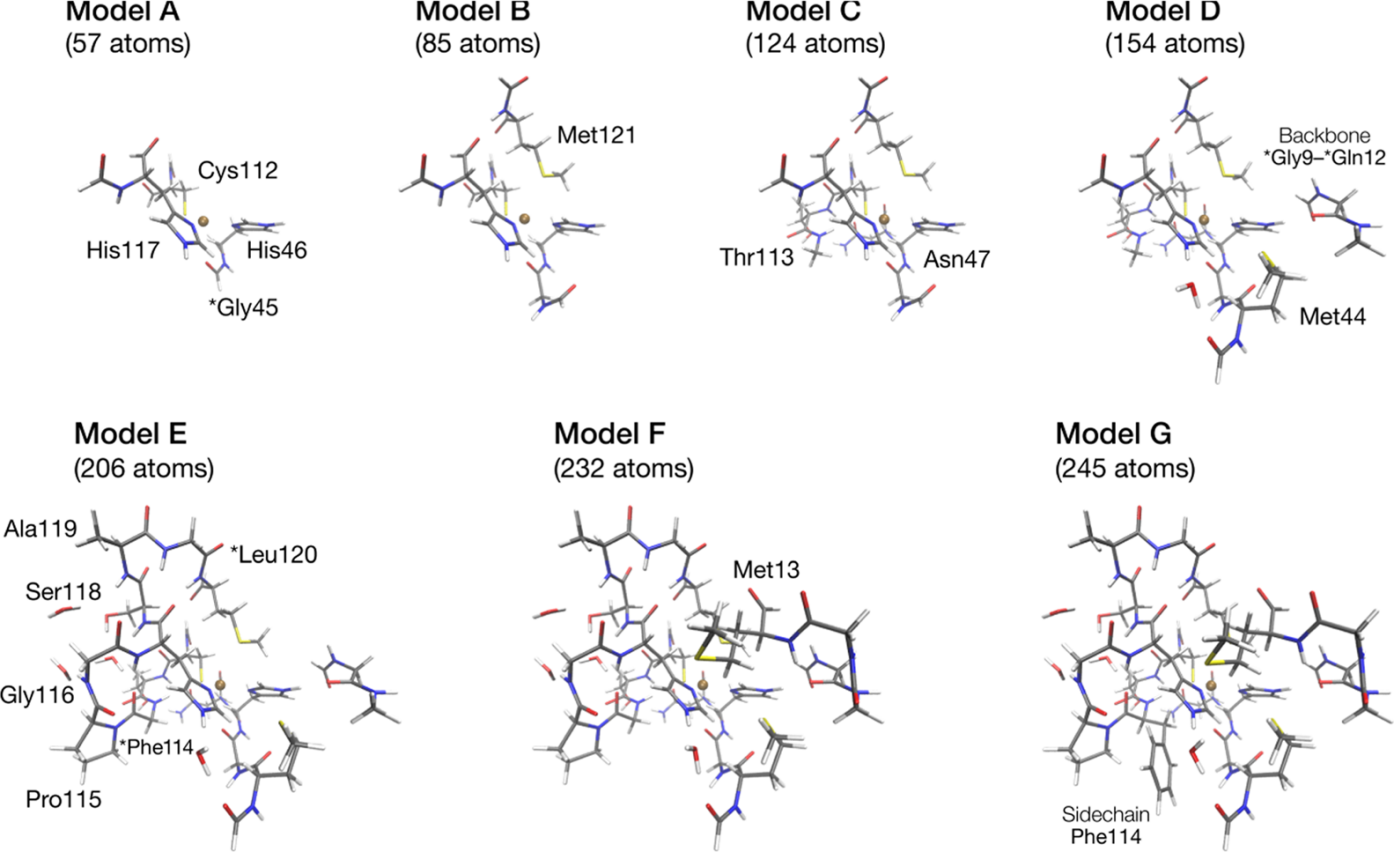
QM regions used in this study with labels indicating the residues included with respect to the previous model. The asterisk indicates that only the backbone of the residue is included.

QM/MM calculations are performed using electrostatic embedding, with linking hydrogen atoms to saturate the QM area. A set of models between 57 and 245 atoms were used, to study the changes of properties with increasing system size. The number of QM atoms was systematically increased by adding functional groups to the model, as shown in Figure 2. The smallest model (57 atoms, model A) only includes copper, the cysteine Cys112 ligand, both histidine ligands His46 and His117, and the glycine Gly45 backbone. Model B (85 atoms) additionally includes the methionine ligand Met121 and an extension to the Gly45 backbone. Adding this backbone extension separately has been tested, but does not affect geometry or electronic structure. For model C, residues which form hydrogen bonds to Cys112 were added. The inclusion of Thr113 and Asn47 leads to a model with 124 atoms. In model D (154 atoms), hydrogen bonds from both histidine ligands were added, which leads to inclusion of the backbone of Gly9 to Gln12 and Met44 with a connecting H_2_O. In model E, the backbone connecting Cys112 and Met121 is added, leading to 206 atoms. To complete the second sphere around the Cu center, an additional methionine Met13 was added (model F, 232 atoms). In the final model (model G, 245 atoms), the so far omitted side chain of Phe114 is explicitly included in the QM part. Further increases in the QM size were not attempted, since these would result in inclusion of a large number of explicit water molecules beyond the protein surface. This would require conformational sampling to a point where size/functionality–property relations can no longer be unambiguously determined.

### 2.2 Computational Details

All calculations were performed with the ORCA program package. QM/MM calculations used an interface of ORCA with NAMD.^55^ DFT calculations used D3BJ dispersion corrections.^56^ Relativistic effects were included with the use of the DKH2 Hamiltonian.^57−60^ It is noted that the ZORA Hamiltonian^61−63^ was also tested and no significant differences were observed from DKH2 in structural parameters. Appropriately recontracted DKH-def2-TZVP(-f) basis sets^64,65^ were used for the DFT calculations, along with decontracted def2/J basis sets^66^ for the RI approximation to the Coulomb integrals. The chain of spheres approximation (COSX)^67^ was used for the exchange. Grids were increased to “Grid5” and “GridX5” in ORCA nomenclature. QM gas-phase optimizations were done using the same settings, with Cartesian constraints on the Cα atoms.

DLPNO-CCSD(T) energies were calculated using TightPNO thresholds on top of a TPSSh^68^ reference. A DKH2 Hamiltonian was employed, with DKH-def2-TZVP basis sets on everything except copper, for which DKH-def2-TZVPP is used. The auxiliary basis sets were generated using the Autoaux feature.^69^ DKH2 was employed with a finite nucleus using a Gaussian core model.^70^ Picture change effects were accounted for.^71^

HFC parameters from DFT were calculated using the TPSSh functional with the DKH2 Hamiltonian with inclusion of picture change and finite nucleus effects.^70,71^ The DKH-def2-TZVP(-f) basis set was used for all atoms except copper and sulfur. For Cu and S, the basis set employed was the DKH-def2-TZVP basis with fully decontracted s-functions and three additional s-functions created by scaling the tightest exponent of the original basis set by 15.625, 6.25, and 2.5.^72^ No RI approximation was used in the calculation of spectroscopic properties. To calculate HFCs from DLPNO-CCSD densities, a previously established protocol was used^51^ that utilizes very tight thresholds for the PNO generation and decontraction of the basis sets. In addition, the multifragment approach within the DLPNO framework is used,^73^ with different thresholds for the PNO generation in the outer fragments. Here, thresholds vary from LoosePNO to TightPNO. Unrelaxed densities were used for the spectroscopic properties derived from the DLPNO-CCSD calculations.

Simulations of EPR and ENDOR spectra were performed using the Easyspin program.^74^ Simulations of the EDNMR spectra were performed with a home-developed program by the Goldfarb lab that was already used for the interpretation of the experimental spectra.^27^

## 3 Results

### 3.1 Geometric Parameters

Since the crucial parameter to the Cu–SCys112 interaction is the distance between them, we require our computational models to yield a Cu–S distance close to experiment. In a first step, the experimental values for the copper ligand distances are analyzed (Table 1 and Figure S1). The X-ray diffraction (XRD) structures show a large variety in the active-site distances. Possible reasons are the empirical restraints used in the refinement of the structure, differences in their resolution, or photoreduction during data collection. In the earliest crystal structure, the Cu–SCys112 distance was deduced to be 1.79 Å, which is unrealistically short. In subsequent XRD models, the distance varies between 2.12 and 2.27 Å. XRD has problems in showing the position of sulfur atoms close to electron-rich atoms, like copper. Here, EXAFS measurements helped to more reliably refine the distance to 2.12 Å.^35,36^ The Cu–N_His_ distances vary similarly, between 1.94 and 2.11 Å for His117, and 1.93 and 2.15 Å for His46. However, there is an observed difference between the two Cu–N_His_ distances in each protein. The Cu–SMet121 distance is much longer than the Cu–SCys112 distance, but much better resolved. The Cu–O distance on the other hand varies between different structures.

**Table 1:** Distances (Å) Between Cu and Surrounding Atoms in the Reduced State of Azurin as Obtained from Structural Studies Reported in the Literature

	Cu–SCys112	Cu–N_δ_His117	Cu–N_δ_His46	Cu–SMet121	Cu–OGly45
1AZU^6^	1.79	2.42	2.15	3.21	2.47
4AZU^52^	2.27	2.11	1.99	3.18	2.84
	2.27	1.98	2.06	3.16	2.95
	2.24	1.96	2.13	3.21	3.05
	2.17	2.00	2.12	3.05	3.03
1DZ0^75^	2.16	2.02	2.03	3.26	2.75
1NWO^76^	2.13	1.94	2.01	3.01	3.16
	2.14	1.96	1.93	3.14	2.95
2CCW^77^	2.21	2.00	2.02	3.26	2.94
2AZA^78^	2.12	2.01	2.08	3.12	3.16
	2.17	1.99	2.09	3.10	3.09
XRD AVG	2.15	2.03	2.05	3.16	2.93
XRD STD	0.12	0.13	0.06	0.08	0.20
EXAFS^36^	2.12	1.86/1.94	1.86/1.94	3.39	2.82

Calculated distances depend on model used and the type of calculation: gas-phase DFT optimizations show the shortest Cu–S_Cys_ distances.^77^ For QM/MM optimization, it depends on the type of coupling and embedding used.^31,77^ The error of DFT was quantified in large-scale QM/MM calculations.^28^ A summary of previous geometries obtained by computational modeling is given in Table S1. We initially tested a few commonly used density functionals for model B (for details, see Supporting Information Table S2). Based on the Cu–S_Cys_ distance as a main criterion, BP86, TPSS,^68^ and TPSSh^79^ were considered. The results are collected in Table 2. Additionally, we tested B3LYP,^80^ because it was used in previous studies.^28^ These functionals were tested against DLPNO-CCSD(T) energies (with DLPNO-CCSD(T) geometry optimizations are not feasible). The results show that TPSSh provides the structure with the lowest DLPNO-CCSD(T) energy, and hence this functional was used in further studies.

**Table 2:** Evaluation of Optimized Geometries Obtained with Different Functionals Showing the Cu–SCys112 Distance and the Relative DLPNO-CCSD(T) Single Point Energies^a^

	Cu–S (Å)	Δ*E* (kcal/mol)
BP86	2.16	2.55
TPSS	2.16	0.81
B3LYP	2.18	0.61
TPSSh	2.16	0

a The TPSSh geometry provides the lowest DLPNO-CCSD(T) energy.

In Table 3, the active-site distances computed with TPSSh for the model series are presented. The Cu–S_Cys_ distance decreases with increasing model size. Still, the EXAFS distance of 2.12 Å is not reproduced. Inclusion of dispersion effects in the form of D3 corrections^56^ leads to slightly shorter distances. The latest D4 dispersion correction^81^ was also tested, but did not change these results in any significant way. The influence of dispersion was additionally assessed by decomposition of the DFT energies. This indicated that the dispersion energy is about 1 order of magnitude smaller than the QM–MM interaction energy. Therefore, in geometry optimizations, the treatment of the environment by the QM/MM approach is much more significant than the inclusion of empirical dispersion corrections.

**Table 3:** Cu–Ligand Distances (in Å) from QM/MM Optimized Models Compared to Averaged EXAFS Distances

	Cu–SCys112	Cu–N_δ_His117	Cu–N_δ_His46	Cu–SMet121	Cu–OGly45
A	2.18	1.94	1.94		2.75
B	2.17	1.94	1.94	3.05	2.91
C	2.16	1.93	1.93	3.09	2.88
D	2.16	1.90	1.91	3.09	2.88
E	2.15	1.89	1.91	3.00	2.95
F	2.14	1.89	1.90	3.07	2.95
G	2.13	1.87	1.90	3.10	2.90
EXAFS^36^	2.12	1.90	1.90	3.39	2.82

Looking at the evolution of all Cu–ligand distances (Table 3), it is found that also the Cu–N_His_ distances decrease with increasing QM size. The weakly bound ligands Met121 and the backbone of Gly45 however vary, depending on the steric hindrance that is included in the respective model.

While the distances show a limited picture, the analysis of angles (Table S3 and Figure S2) allows us to obtain a complementary view of the changes at the copper center upon increasing model sizes. While the Cu–S_Cys_ distance decreases, the C_α_–C_β_–S_Cys_ angle, which defines the position of the side chain with respect to the backbone, remains constant. However, the C_β_–S_Cys_–Cu angle, which was found crucial for description of the Cu–S_Cys_ interaction,^16,37^ decreases with increasing model size. With increasing model sizes, also the Cu–N_His_ distances decrease. The corresponding angle between the His117 N_ε_–N_δ_–Cu decreases, which describes a movement of the imidazole group of His117 toward the copper center. Such a movement is not observed for His46. Instead, the dihedral angle between the imidazole plane and the Cu–S_Cys_ vector decreases. This means that the imidazole group of His46 moves out of the plane defined by Cu, S_Cys_, and N_ε_ of His46. To compare this behavior to experimental geometries, the distances from the QM/MM optimized models are compared to the crystal structure averages in Figure S3. The Cu–S_Cys_ distances are within the range of distance reported in various crystal structures. However, for these first coordination shell distances, EXAFS is presumably providing more accurate numbers. Here, the EXAFS data give an average Cu–N_His_ distance of 1.90 Å.^36^ The QM/MM results are in good to excellent agreement with this result. The QM–MM interaction was found to affect the Cu–SCys112 distance. Scans along the Cu–S_Cys_ coordinate indicate that neglect of the QM–MM interaction energy while maintaining the QM/MM structural constraints shifts the minimum by 0.05 Å toward longer distances. Interestingly, QM-only cluster optimizations that completely neglect the protein environment also lead to short Cu–S_Cys_ distances (Table S4) in agreement with previous gas-phase calculations,^77^ but this is accompanied by other structural changes within the copper coordination sphere that are not consistent with the QM/MM geometries. Therefore, sufficient treatment of the environment in the QM/MM approach is essential for the description of the geometry of the copper site. These QM/MM structures were used as the basis for the calculation of spectroscopic parameters discussed in the following.

### 3.2 Hyperfine Coupling Constants

In the following, we present calculations of all hyperfine parameters that are relevant for understanding the electronic structure of the copper site and for establishing connections to experimental observations. We employ the highest level of theory available to us, DLPNO-CCSD. To evaluate the DLPNO-CCSD results, we present simulated spectra obtained with the calculated parameters and compare to experiment. This allows us to evaluate whether the calculated spin distribution over the active site, and in particular over copper and sulfur centers is accurate. Comparing the calculations with all experimental hyperfine information will allow us to develop a more global picture of the spin distribution and pinpoint where the calculations may fall short. The final outcome will be calibrated spin populations (in a given population analysis scheme) that may serve as reference for other theoretical methods. In the Supporting Information, we also provide a comparison with hyperfine couplings and spin populations from DFT.

The calculation of HFCs using DLPNO-CCSD was tested in a benchmark study for various molecules including transition-metal complexes by Saitow et al.^51^ Due to the size of the azurin models, a multifragment approach was utilized.^73^ This ensures to keep the accuracy of the established protocol (for a test on model A, see Table S5) but exceed its limits regarding the model size. In a two-fragment scheme, the atoms for which HFCs will be computed are included in fragment 1. For these atoms, the required tight thresholds are used.^51^ The rest of each model is included in fragment 2. Using LoosePNO settings for fragment 2, the HFCs of all model sizes up to model G can be calculated. In Table 4, the results for the largest HFCs are shown.

**Table 4:** Model A to G DLPNO-CCSD Hyperfine Coupling Constants (MHz)^a^

	SCys112	N_δ_His117	N_δ_His46	H_A_	H_B_
A	19.6	23.8	21.3	21.6	9.0
B	19.1	24.6	20.8	17.8	13.7
C	19.4	24.9	21.8	17.3	14.8
D	15.6	31.6	21.3	10.6	12.5
E	14.2	32.7	22.1	9.5	10.5
F	14.8	32.4	22.2	10.3	11.0
G	15.2	32.8	22.1	10.4	11.9

a Tight thresholds were applied in fragment 1 as defined in the text; LoosePNO thresholds were applied in fragment 2. A graphical representation is given in Figure S4. A similar table with the principal values is provided as Table S6.

This approach allows access to all sizes for the QM subsystem, but the drawback of the two-fragment scheme is the discontinuity between HFC atoms and the amino acid side chain that can lead to an incorrect description of spin delocalization. This can be observed, for example, in the case of HFCs of the cysteine β-hydrogens, but is also expected for the histidine ring. Therefore, we adopted instead a three-fragment scheme, where the amino acid side chain of each ligand is treated at an intermediate level of accuracy (fragment 2), and the rest of the model is treated as a lower-threshold fragment 3. This improves the quality of the ligand description (see Table 5) albeit leading to increased computational cost, which means that not all model sizes can be treated this way. The best compromise between model size and accuracy was found for QM/MM model C. The corresponding Mulliken and Löwdin spin populations for model C are given in Table S7.

**Table 5:** Model B/C DLPNO-CCSD Hyperfine Coupling Constants (MHz)^a^

	SCys112	N_δ_His117	N_δ_His46	H_A_	H_B_
B (tight/loose)	19.9	24.8	20.7	22.3	17.4
C (tight/loose)	20.1	25.2	21.8	21.2	18.5

a Tighter thresholds in fragments 1, 2, and 3 as indicated.

#### 3.2.1 *g*-Tensor and Copper Hyperfine Coupling

Although the focus of our work is the ligand HFCs and their comparison to experiment, we briefly discuss the question of the *g*-tensor of the system and of the copper HFC. The *g* tensor is needed to define the orientation of the HFC tensors since the eigensystem of ***g***^T^ ***g*** serves as the reference frame for the EPR simulations. As observed previously^32^ and also shown in Figure S5, the calculated *g* tensor and copper HFC deviate from experiment. This is a known limitation of currently available theoretical approaches. Particularly, the copper HFC is one of the most challenging quantities to compute accurately, regardless of the level of quantum chemical approximation.^32,46,82^ Therefore, to avoid any ambiguity, the experimental *g* values are used in the following, whereas the orientation is taken from the DFT calculations. It has generally been found to be in good agreement for the case of plastocyanin, where the *g*-tensor orientation had been deduced from single-crystal EPR experiments.^4,46^ The orientation of the *g* tensor in the molecular frame is shown in Figure S6. The agreement of the calculated HFCs with experiment is shown by the comparison between simulated spectra using the calculated ligand HFCs and the experimental ENDOR^83^ and EDNMR^27^ experiments. By directly comparing simulated and measured spectra, we avoid any misinterpretation that may arise from HFC parameters deduced from simulating the experimental spectra.

#### 3.2.2 Nitrogen Hyperfine Couplings

We consider four nitrogen atoms, the coordinating δ nitrogens of the histidines His117 and His46 and the remote ε nitrogens of the imidazole ligands. The remote nitrogen HFCs are of the order of 1 MHz,^21^ which is perhaps too small to be analyzed with confidence given the intrinsic uncertainties of the computational method.^51^ Nevertheless, the calculated values, 1.2 MHz for N_ε_His117 and 1.0 MHz for N_ε_His46, accurately reproduce the experimental observations, 1.3 MHz for N_ε_His117 and 0.9 MHz for N_ε_His46, as deduced from electron spin echo envelope modulation (ESEEM) experiments.^21^

The HFCs of the coordinating histidine δ-nitrogens are shown in Table 6. The N_δ_His117 HFCs agree very well with the ESEEM *A*_iso_, but are smaller than the EDNMR results. Given the limited accuracy of the EDNMR results, the calculated N_δ_His117 HFCs show reasonable agreements. The N_δ_His46 HFC is about 1 MHz larger than the ESEEM value, but within the range of the EDNMR values. Again, for the EDNMR HFCs, a larger error is observed than for the ESEEM HFCs. This difference in the error might stem from the fact that the ESEEM was measured on a single crystal,^21^ while the EDNMR data were obtained from frozen solution samples. A close-up on this issue is presented in the simulation of the experimental spectra below.

**Table 6:** Components of the N_δ_His HFCs Calculated with DLPNO-CCSD from Model C Compared to the Experiment^a^

N_δ_His117	*A_xx_*	*A_yy_*	*A_zz_*	*A*_iso_	N_δ_His46	*A_xx_*	*A_yy_*	*A_zz_*	*A*_iso_
DLPNO-CCSD	29.4	22.7	23.4	25.2	DLPNO-CCSD	25.7	19.6	20.2	21.8
ESEEM^21^	27.8 (±0.4)	24.0 (±0.3)	23.6 (±0.3)	25.1 (±0.3)	ESEEM^21^	19.1 (±0.3)	18.0 (±0.4)	17.2 (±0.4)	18.1 (±0.4)
EDNMR^27^	32.8 (±1.5)	25.0 (±1.5)	24.5 (±1.5)	27.4 (±1.5)	EDNMR^27^	24.0 (±0.8)	21.0 (±0.8)	17.8 (±0.8)	20.9 (±0.8)

a Note that notation was adjusted to align with experiment, as *A*_*xx*_ is the largest component of the HFC.

Along with the δ-nitrogen HFCs, the underlying interactions can be analyzed by decomposition of the HFC onto the multicenter components (Table 7). Here, it is shown that the one-center terms, that is, the local contributions, are dominant among the other interactions. This indicates that the spin density can be approximately assigned to individual atoms. Hence, one can attempt to correlate a given HFC with an atomic spin population value. This is done here using the Mulliken population analysis scheme, which leads to DLPNO-CCSD spin populations of 0.0532 for N_δ_His117 and 0.0501 for N_δ_His46.

**Table 7:** Decomposition Analysis for the Nitrogen Hyperfine Coupling Constants

N_His117_	*A*_min_	*A*_mid_	*A*_max_	N_His46_	*A*_min_	*A*_mid_	*A*_max_
1-center	1.68	0.63	–2.31	1-center	0.98	0.82	–1.80
2-center pc	0.49	0.28	–0.77	2-center pc	0.29	0.38	–0.67
2-center bond	–0.25	–0.18	0.43	2-center bond	–0.14	–0.22	0.37
3-center	–0.03	0.01	0.02	3-center	–0.01	–0.01	0.02
total	1.89	0.73	–2.62	total	1.12	0.97	–2.08

**Figure 3:**
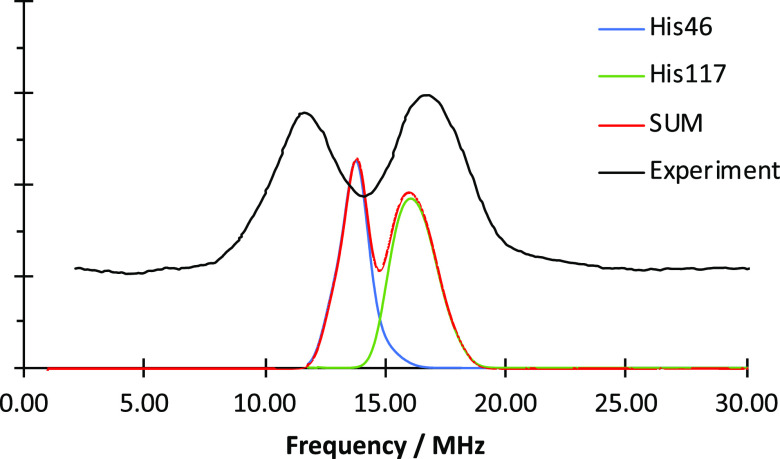
Comparison of the ^14^N ENDOR simulation with hyperfine and quadrupole coupling constants obtained from DLPNO-CCSD as described in the text (red line), compared to the simulation using parameters determined from experiment^83^ (black dotted line) and the experimental ENDOR spectrum^84^ (black line). Experimental parameters: *ν* = 35.2 GHz, *B* = 1113 mT.

The comparison with the ENDOR simulation is shown in Figure 3. In the experimental Rapid Passage ENDOR, the majority of the intensity lies in the signals corresponding to , while the  transition is almost not detectable. The signals of His117 show good agreement with experiment, while the signals of His46 are slightly too high in energy. The calculated HFC for N_δ_His46 is 21.8 MHz, while experiment shows an HFC of 18.1 MHz. At the same time, the width of the signal deviates from experiment, which could arise from inhomogeneous broadening, or differences in the orientation of the calculated quadrupole interaction.

**Figure 4:**
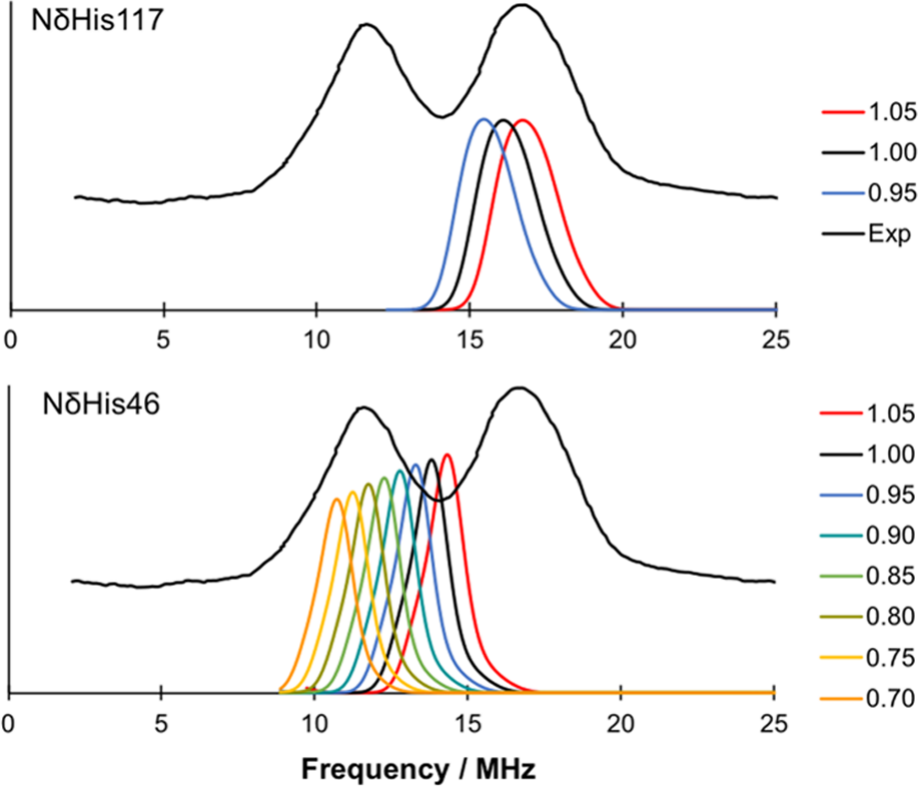
Scaling of the calculated DLPNO-CCSD HFCs for the N_δ_ of His117 (top) and His46 (bottom).

To evaluate the agreement between the DLPNO-CCSD ^14^N HFCs and experiment in a more quantitative way, the DLPNO-CCSD HFCs are scaled in 5% increments. The results are shown in Figure 4. For the N_δ_His117 HFCs (top), the best agreement is observed if 5% of the value is added to the DLPNO-CCSD values. This corresponds to an HFC of 26.4 MHz, which is roughly midway between the experimental values obtained by Coremans et al.^21^ and by the Goldfarb group.^27^ Reducing the N_δ_His117 HFC on the other hand leads to a clear disagreement with experiment. For the N_δ_His46 HFC (Figure 4 bottom), a more complicated picture is observed. The best agreement with experiment is observed if the DLPNO-CCSD *A*_iso_ is reduced by 20%. This corresponds to an HFC of 17.5 MHz, which is closer to the Coremans et al. value of 18.1 MHz.^21^

Given this difference between the DLPNO-CCSD HFC and the scaled HFC, one might wonder how this translates back to the spin population on the δ-nitrogens. The DLPNO-CCSD Mulliken spin population on the δ-nitrogen of His117 is 0.0532, and after scaling, it is 0.0558. On His46, the DLPNO-CCSD calculation yields a spin population of 0.0501, but after scaling the HFC down by 20%, only 0.0400 of the spin is found on the δ-nitrogen of His46. These numerical differences are not large, yet the simulations demonstrate that the spectra are sensitive to subtle differences of this magnitude. These differences can arise from slight variations in the geometries, such as a small rotation of the imidazole ring. Hence, high-accuracy calculations are needed to describe the system properly.

**Figure 5:**
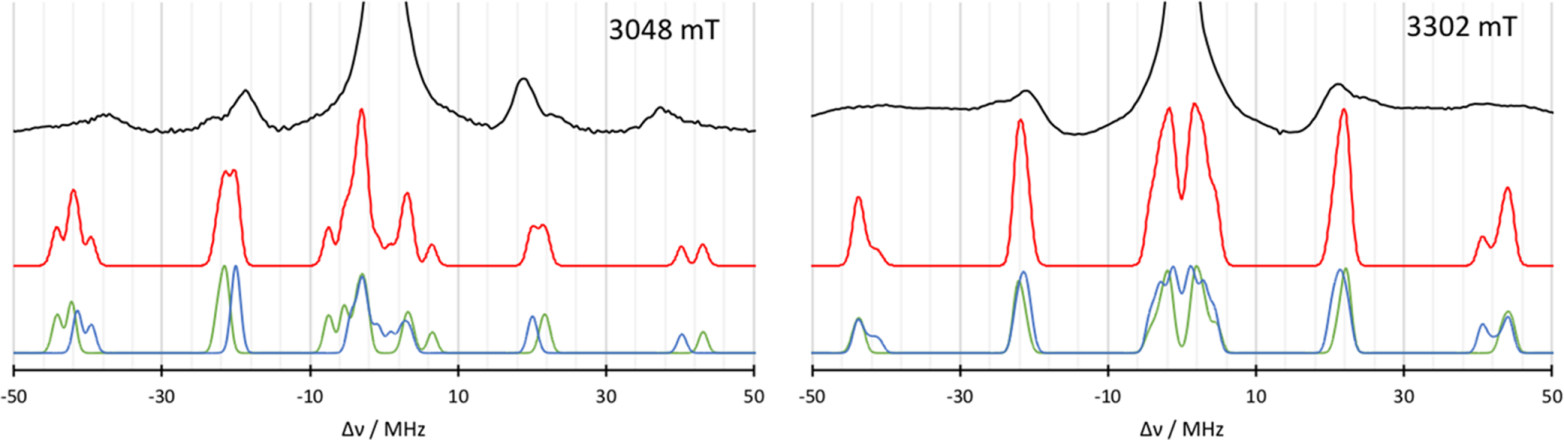
Comparison of the simulated EDNMR spectra obtained from QM/MM calculations (red) with the subspectra of N_δ_His46 (blue) and N_δ_His117 (green) for 3048 mT (left) and 3302 mT (right) to the experiment^27^ (black lines). Microwave frequency *ν* = 94.9 GHz.

**Figure 6:**
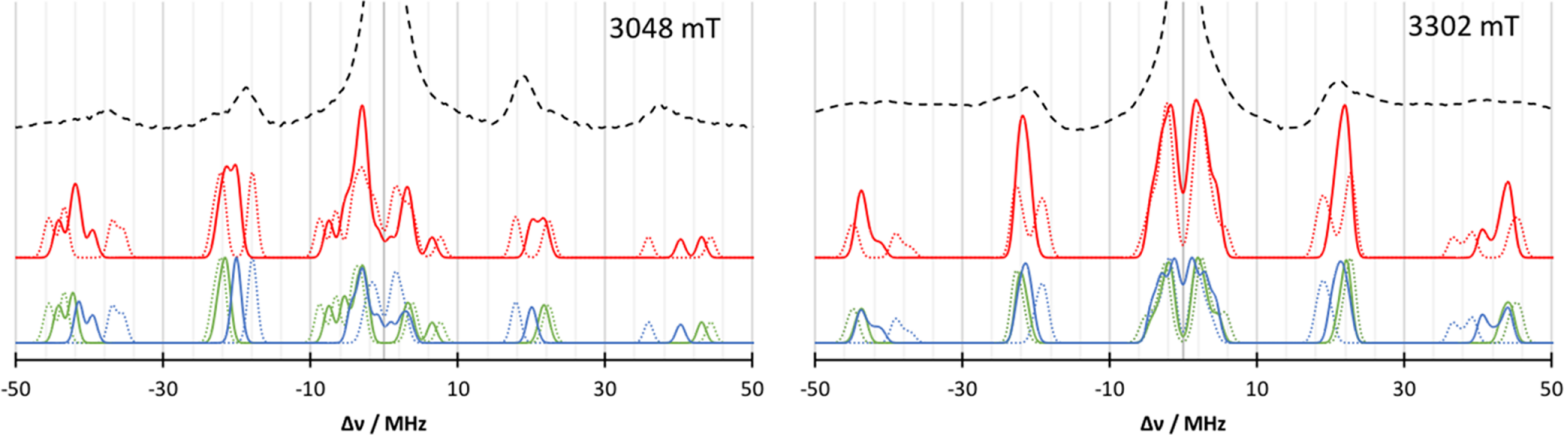
Comparison of the simulated EDNMR spectra obtained from QM/MM calculations (red) with the subspectra of N_δ_His46 (blue) and N_δ_His117 (green) for 3048 mT (left) and 3302 mT (right) to the experiment^27^ (black lines). Dotted lines indicate scaled Nitrogen HFCs according to the ENDOR discussion before. Microwave frequency *ν* = 94.9 GHz.

The second spectrum that can be used for comparison is the EDNMR of unsubstituted azurin. The simulation for the EDNMR spectrum at two different magnetic fields is given in Figure 5. Again, the subspectra of the individual histidines are shown. In both spectra, the general features below 30 MHz are reproduced very well. At 3048 mT, the calculated spectrum is narrower around the 20 MHz area than the experimental spectrum and a shoulder at 25 MHz is missing, which could be attributed to the differences between computed HFCs and experiment. The calculated N_δ_His46 HFCs of 21.8 MHz are slightly larger than the experimental results of 18.1 MHz^21^ or the refitted value of 20.1 MHz.^27^ While the DLPNO N_δ_His117 HFCs of 25.2 MHz agree very well with the ESEEM results,^21^ they were refitted to 27.2 MHz in the EDNMR.^27^ However, in the 40 MHz region, the calculated signals are visibly higher than the experimentally observed signals, i.e., both HFCs disagree. Since this signal is due to double quantum transitions, a twice large discrepancy is to be expected. Additionally, contributions from ^63,65^Cu lead to a broad signal around 40 MHz, which leads to difficulties in the interpretation. A similar picture is given by the simulated spectrum at 3302 mT, where the signals observed at around 45 MHz in the calculated spectrum cannot be found in the experiment. Again, a broad copper band situated between 30 and 50 MHz is masking the signals. To test the effect of the scaling, the results of the ENDOR comparison are used in EDNMR simulations in Figure 6. In the 3048 mT spectrum, the scaled nitrogen HFC of N_δ_His46 provides a too early onset in the 16 MHz region. In the −18 MHz region, however, the scaling improved the agreement with experiment. In the region beyond ±30 MHz, it is difficult to judge whether the scaling leads to an improvement. In the 3302 mT spectrum, the onset of the ±16 MHz signals has been improved by scaling the HFC values. Here again, the N_δ_His46 HFC is the main origin for the improvement. Similar to the 3048 mT spectrum, the region beyond 30 MHz cannot be judged due to the broad experimental lines.

#### 3.2.3 Proton Hyperfine Couplings

The proton HFCs can provide a complementary perspective on the electronic structure of the copper site. We first look at the HFCs of imidazole ring protons of histidines His46 and His117, which are of the order of 1 MHz.^22^ The DLPNO-CCSD results are given in Table 8. Although these values are small, there is convincing correspondence of the computed values with those deduced from NMR experiments.^22^

**Table 8:** DLPNO-CCSD Hyperfine Coupling Constants (in MHz) of the Histidine Ring Protons, Compared to Experiment

	N_ε_H(His117)	C_ε_H(His117)	N_ε_H(His46)	C_ε_H(His46)
DLPNO-CCSD	1.0	0.8	1.0	0.9
NMR^22^	1.1/1.5	0.56	1.1/1.5	0.56

**Figure 7:**
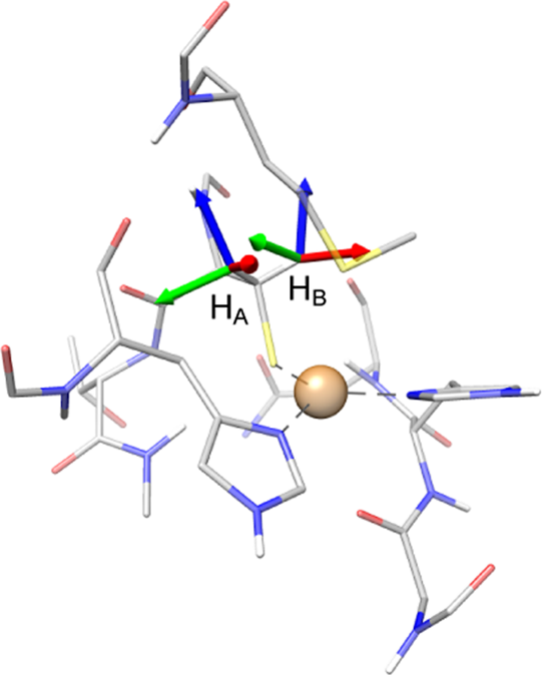
Orientation of the Cys112 β-proton HFC tensors at the azurin T1 center. Directions: *z* in blue, *y* in red, and *x* in green.

The situation with the two cysteine β-protons, experimentally termed H_1_ and H_2_, is more complicated. In contrast to the histidine ring protons, selective isotope substitution is not possible for the protons on the cysteine β-carbon. Hence, while it is possible to determine individual HFC values for each β-proton, no clear assignment to the individual atoms was possible.^22−24^ However, orientation-dependent ^1^H ENDOR measurements provide information on the orientation of the *A* tensor of each cysteine β-proton.^25^ Using this information, a direct assignment with the calculated proton HFCs is possible, by comparing the calculated orientations to the HFC orientation determined experimentally (cf. Figure 7).^25^ From this analysis, it can be deduced that DLPNO-CCSD H_A_ corresponds to ^1^H-ENDOR H_2_,^25^ and vice versa (H_B_ to H_1_). Looking at the absolute values of the HFCs for the cysteine β-protons (Table 9), this assignment seems opposite to what is suggested numerically, but given the possible error of the DLPNO-CCSD method and experimental uncertainties, the directions of the principal axes of the hyperfine tensor are probably more reliable in this case, and therefore we suggest that the correspondence of nuclei established from the tensor orientations is more reliable as well.

**Table 9:** DLPNO-CCSD Hyperfine Coupling Constants (in MHz) of the Cysteine β-Protons, Compared to Experiment

	*A_xx_*	*A_yy_*	*A_zz_*	*A*_iso_
DLPNO-CCSD H_A_	18.7	19.8	25.0	21.2
DLPNO-CCSD H_B_	15.9	17.1	22.6	18.5
ENDOR^25^ H_1_	20.4	21.3	26.2	22.6
ENDOR^25^ H_2_	14.4	19.1	23.0	18.8

**Figure 8:**
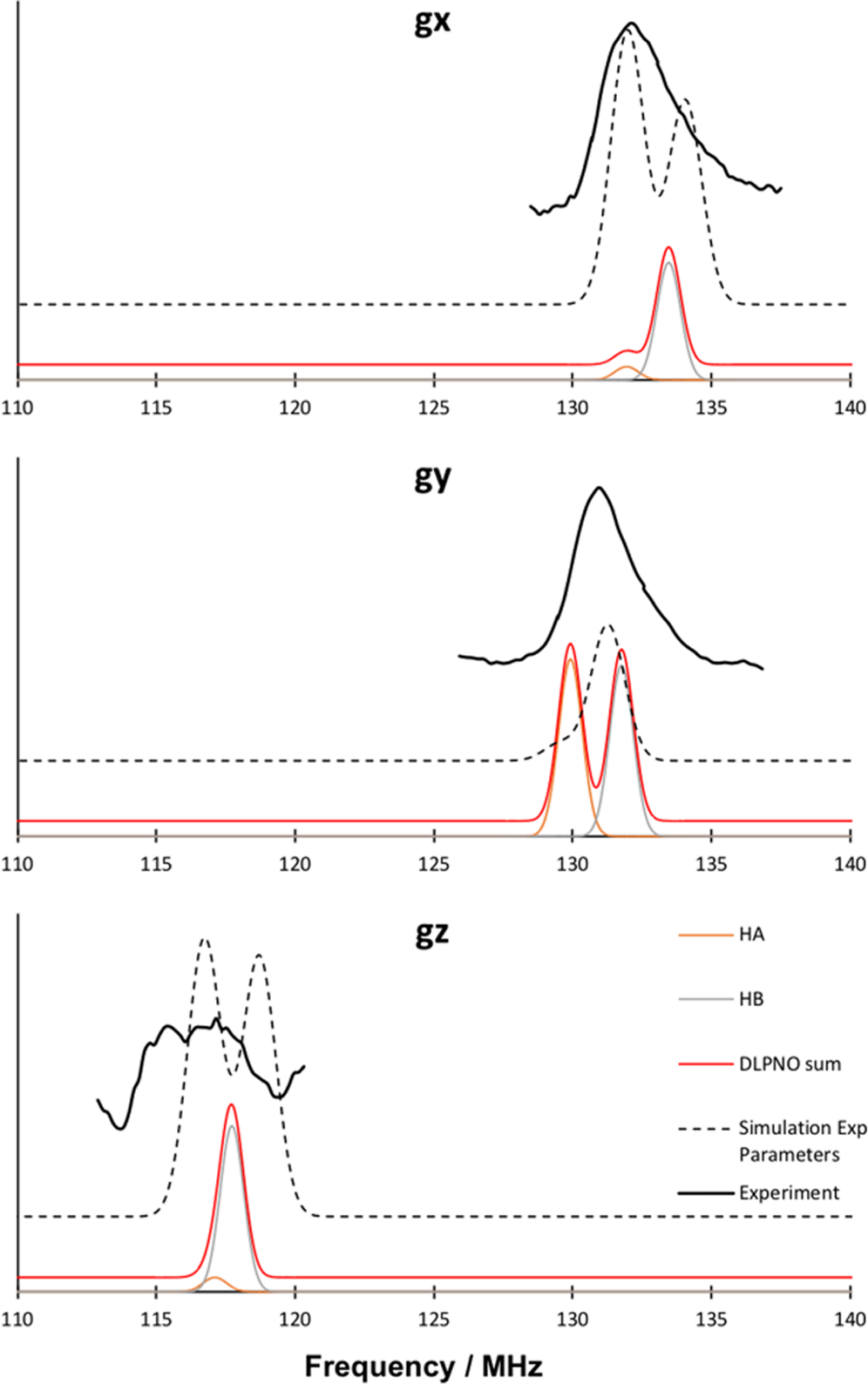
Comparison of the simulated ^1^H ENDOR using the DLPNO-CCSD parameters (red line), consisting of the H_A_ (orange) and H_B_ (gray) subspectra with experiment^25^ (black full lines) and simulation of the experimental parameters (black dashed lines), for the individual directions of the *g* tensor. Field of 3328.69 mT (*g*_*x*_, *ν*_Zeeman_ = 141.73), 3300.37 mT (*g*_*y*_, *ν*_Zeeman_ = 140.52), and 3016.98 mT (*g*_*z*_, *ν*_Zeeman_ = 128.46). Microwave frequency: *ν* = 94.9 GHz.

The proton ENDOR spectrum can similarly be simulated and compared to experiment (Figure 8). In general, a very good agreement is observed between experiment and the spectrum obtained on the basis of DLPNO-CCSD computed values. In the *g*_*x*_ direction, perfect agreement is observed for the H_A_ subspectrum, although it is not as intense as experiment. H_B_ agrees very well with experiment, especially considering the simulation of the experimental parameters. In the *g*_*y*_ direction, again good agreement is observed. Here, the signals of the DLPNO-CCSD simulation are clearly separated, while the experiment is convoluted in a single peak. In the *g*_*z*_ direction, the opposite is observed. Here, the DLPNO-CCSD parameters of the individual protons overlap, while the experiment shows a wider signal with two individual peaks. These slight disagreements might originate from differences in the orientation of the tensor, in combination with the position of the β-protons in the model.

At this point, the origin of the HFC can be discussed. As shown from the decomposition of the HFC (Table 10) in the multicenter parts, the majority originates from the two-center nonbonding interaction, which translates to the dipolar terms. Hence, a translation back to the spin population on the β-hydrogens as done for the δ-nitrogens does not make sense. These small differences clearly show that accurate methods are needed to determine the electronic structure and also that small differences in the geometry could dramatically change the results in this case.

**Table 10:** Decomposition Analysis for the Cys112 β-Proton Hyperfine Coupling Constants

H_A_	*A*_min_	*A*_mid_	*A*_max_	H_B_	*A*_min_	*A*_mid_	*A*_max_
1-center	0.03	–0.06	0.04	1-center	0.04	–0.06	0.02
2-center pc	1.64	1.98	–3.62	2-center pc	2.04	2.27	–4.32
2-center bond	0.37	–0.50	0.13	2-center bond	0.41	–0.52	0.10
3-center	–0.63	–0.51	1.15	3-center	–0.64	–0.59	1.22
total	1.40	0.91	–2.31	total	1.86	1.11	–2.96

#### 3.2.4 ^33^S Hyperfine Couplings

A crucial spectroscopic quantity is the sulfur HFC, which has been limited by the applicability of ^33^S labeling to the Cys112 ligand. Recently, a 1D-EDNMR spectrum was recorded, where the sulfur HFC could be determined.^27^ Unfortunately, the resolution of these 1D-EDNMR spectra is rather limited; hence, 2D-EDNMR spectra were also recorded. In the following, first, we compare the computed HFC values, and then we proceed with simulations of the EDNMR spectra. Unfortunately, the two experimental approaches yield slightly different fitted parameters for the sulfur HFC (Table 11), both for the isotopic value and for the principal values. While the general trends are the same, a larger *A*_*zz*_ value but smaller *A*_*yy*_ value is observed for the 1D-EDNMR. The calculated ^33^S HFCs show good agreement with the 1D-EDNMR experiment, but yield an even larger *A*_iso_. Hence, the agreement with the fits from the 2D-EDNMR is rather limited.

**Table 11:** ^33^S Principal HFC Values (MHz) from DLPNO-CCSD, Compared to Experiment

sulfur	*A_xx_*	*A_yy_*	*A_zz_*	*A*_iso_
DLPNO-CCSD	–11.5	–13.8	85.5	20.1
1D-EDNMR^27^	–15.4 ± 0.6	–17.0 ± 0.6	89.0 ± 0.6	18.8 ± 0.6
2D-EDNMR^27^	–15.4 ± 1	–27.0 ± 1	67.5 ± 2.5	8.3 ± 1.5

**Figure 9:**
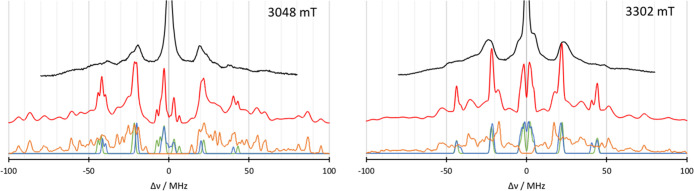
Comparison of the simulated 1D-EDNMR spectra obtained from DLPNO-CCSD calculations (red, broadened to better approximate the experiment) with the subspectra of SCys112 (orange), N_δ_His46 (blue), and N_δ_His117 (green) for 3048 mT (left) and 3302 mT (right) to the experiment^27^ (black dashes). Microwave frequency: *ν* = 94.9 GHz.

The sulfur HFCs are compared to the EDNMR experiment with ^33^S-labeled azurin.^27^ An overview is given in Figure 9. The ^14^N and ^33^S signals overlap strongly. At 3048 mT, the sulfur transitions are observed as resolved features in 20–30 MHz, next to the nitrogen transitions at 20–25 mT other clear features appear at 40 and 60 MHz. At higher fields, shown in the case of 3302 MHz, the sulfur signals are broader, less resolved, and span the area between 10 and 50 MHz. If the subspectra are summed, a good agreement in the shape of the features is observed. However, the detailed signature of the ^33^S is lacking from experiment, especially at higher energies. At 3302 mT, the calculated spectrum rises below 20 MHz, while the experimental onset is found at slightly higher energies. The interpretation of the experimental spectra, and consequently the comparison to the calculation, is limited due to the complexity of the experiment and the large span of the ^33^S EDNMR signals of up to 100 MHz. Because of this, a unique assignment of the calculated parameters to the experiment is not straightforward.

**Figure 10:**
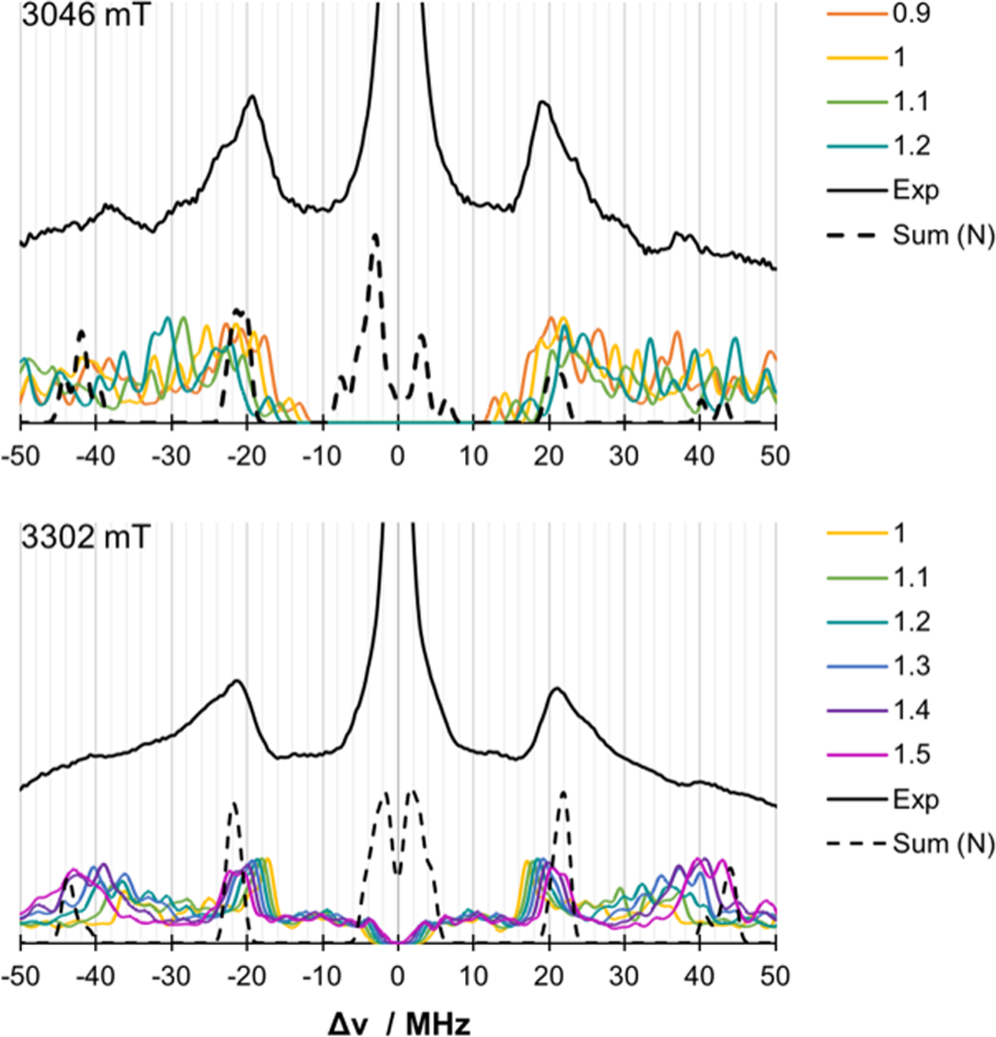
Scaling of the calculated DLPNO-CCSD HFCs for ^33^S at a field of 3048 mT (top) and 3302 mT (bottom) compared to the experiment^27^ (black lines). The sum of the nitrogen spectra (dashed lines) is given as a guide. Microwave frequency: *ν* = 94.9 GHz.

Similarly to the nitrogen HFCs, the sulfur HFCs were scaled and compared to the experiment. As shown in Figure 10, the comparison is not straightforward due to the limited resolution of the experiment. However, looking more closely at the signals around 20 MHz, it becomes obvious that values higher than the calculated sulfur HFCs are not in agreement with the experiment. This is particularly interesting since the original fits show lower HFC values for the sulfur HFC.

To overcome the resolution issues in the 1D-EDNMR, 2D-EDNMR spectra were recorded.^27^ The simulation of the 2D-EDNMR spectra using the DLPNO-CCSD computed parameters was attempted for completeness. The results are discussed in the Supporting Information. As shown in Figure S7, the computed values are in overall agreement with experiment; however, the complexity of the spectra and the uncertainties involved in defining their information content do not permit a detailed analysis in this case.

It is interesting to examine how the above translates to the spin population on the sulfur. The Mulliken spin population obtained from the DLPNO-CCSD calculation is 0.37. The unscaled ^33^S HFCs show a reasonable agreement with experiment, and all scaling tests show that a higher ^33^S HFC leads to better agreement with experiment, which would correlate to a higher spin population. This would then lead to an increased covalency between the copper and sulfur.

Additional insight is offered by the decomposition of the DLPNO-CCSD HFCs into multicenter terms. The one-center term describes the dipole interaction between the nucleus and the spin at atom A. The two-center terms are subdivided into bonded and nonbonded interactions. They describe the dipole interaction between the nucleus of atom A and spin at atoms B and C, bonded and nonbonded respectively. Similarly, the three-center term describes the interaction of the nucleus of atom A and the spin at atom C. The results for the HFC decomposition analysis, presented in Table 12, indicate that the one-center terms are indeed dominating the results for sulfur. However, for a quantitative analysis, the two-center bonded contributions should definitely be included, as they make up about 10% of the final HFC value.

**Table 12:** DLPNO-CCSD HFC Decomposition for ^33^S HFCs (in MHz)

	*A*_iso_	*A*_min_	*A*_mid_	*A*_max_
1-center	21.94	28.97	13.49	–42.46
2-center pc	–0.17	–0.70	0.28	0.42
2-center bond	–1.82	0.01	–0.09	0.08
3-center	0.12	0.04	–0.01	–0.03
total	20.07	28.32	13.66	–41.98

Overall, the above analysis indicates that while the one-center terms are of general importance for the sulfur nucleus, it is not possible to neglect the multicenter terms in the analysis of ^33^S HFCs. Thus, the spin population should be discussed by comparing the experimental HFCs to sufficiently accurate quantum chemical calculations.

## 4 Discussion and Conclusions

In this work, the geometry and electronic structure of the azurin T1 copper center were assessed by a series of computational models. The copper environment was studied with a QM/MM model series of increasing size, to obtain a reasonable copper–sulfur bond distance. The hyperfine coupling constants of ^13^N, ^1^H, and ^33^S nuclei of the copper ligands were computed using the highest level of theory currently available to us, namely, the DLPNO implementation of CCSD. The results and the accompanying analysis demonstrate the need for high numerical accuracy. By simulating the respective spectra using the DLPNO-CCSD parameters, a direct comparison between calculation and experiment is possible. In contrast to the performance of density functional theory (DFT, results presented in the Supporting Information), the calculated DLPNO-CCSD parameters provide meaningful insights into the electronic structure of the azurin Cu(II) center and yield results in good agreement with experiment, or at least within the experimental uncertainty, even though in certain cases, the width and complexity of experimental spectra do not allow highly accurate comparisons and conclusive deductions. Here, spectroscopic studies on well-defined copper model complexes might be of aid to improve the interplay between calculation and spectroscopy. Importantly, we showed that the DLPNO-CCSD method is able to describe the highly covalent Cu–S_Cys_ interaction in the azurin copper center very well by comparing the calculated HFCs directly with experiment instead of relying on empirical relationships.

An important question that has been a central point of contention in the literature on the azurin Cu site is the concept of spin population and the covalency of the Cu–S_Cys_ bond. We note that often the term “spin density” is inappropriately used in experimental works to refer to what is correctly termed “spin population”, i.e., the more or less arbitrary partitioning of the continuous spin density (a global physical quantity) and its assignment to individual nuclei or atoms. From a quantum chemical perspective, there is no unique way of performing this partitioning. Hence, the numerical values depend on the protocol used, for example, the Mulliken or Löwdin population analysis scheme. In fact, from the numbers collected in the Supporting Information (Table S7), the Mulliken and Löwdin spin populations on copper or sulfur deduced from the same many-particle wavefunction differ by up to 6%. This is a strong reminder that these spin populations do not represent physical observables, and if one wants to judge the quality of a given calculation, one should resort to actual physical observables rather than derived quantities like spin populations. Indeed, a large variety of different values for the spin populations in type 1 copper sites have been reported in the literature on the basis of different flavors of DFT (Table S9). If we had to discuss the Cu–S_Cys_ covalency in these terms, our best estimates deduced from fitting the DLPNO-CCSD values to experimental spectra suggest that the average of the two spin population schemes (Table S7) on copper and sulfur amount to 55.3 ± 2.9 and 34.3 ± 2.9%, respectively. For sulfur, this is larger than the 30% suggested by EDNMR,^27^ but possibly slightly smaller than the 37% obtained from XAS.^18,19^ Note that either technique relies on a number of strongly implying assumptions, e.g., one-center approximation in the case of magnetic resonance techniques or reference compound calibrations in the case of XAS. The range of validity of these assumptions has been discussed in the present work and shown to be rather limited. More in-depth discussions can be found in the literature for XAS^85−87^ and magnetic resonance spectroscopies.^33,34,88^

Despite the fact that the present study perhaps represents the theoretically most rigorous attempt to compute metalloprotein active site spectra, there is still ample room for improvement on the computational side, since neither the structural models that are used to represent the active site nor the theoretical method itself are perfect. For example, the DLPNO-CCSD framework for the calculation of hyperfine coupling constants currently does not include triple excitations, nor can it directly address at the moment the second-order spin–orbit contribution to the hyperfine interaction.^51^ Yet, we have shown that it is possible to obtain a very accurate electronic structure picture of all ligand atoms surrounding the azurin copper site. We have also demonstrated what is in our view the best possible use of the raw computed values, that is, the simulation of the spectra and the direct comparison with experimental spectra. Therefore, the DLPNO-CCSD method can be of great assistance in the interpretation of highly complex experiments, and we hope that the present study served to demonstrate the principles of such an approach. Further refinements in this line of research are underway, and we expect that they will have wider implications in raising the standards of what is considered as “mainstream” quantum chemical approaches for the EPR spectroscopy of metalloenzymes in the near future.

## Supporting Information Available

Structural analysis of previous experimental and computational models of azurin; selection of DFT method for geometry optimization; convergence of geometric parameters with the size of the QM region; comparison of QM/MM optimized structures with crystallographic data; detailed DLPNO-CCSD results for hyperfine coupling constants; Mulliken and Löwdin spin populations; simulations of Q-band and W-band EPR spectra; orientation of the copper hyperfine tensor with respect to the experimentally determined *g* tensor; calculated quadrupole splittings from DLPNO-CCSD; comparison of simulated and experimental 2D-EDNMR spectra; and hyperfine coupling constants from DFT (PDF)

## References

[ref1] Underwood E. Trace Elements in Human and Animal Nutrition; Elsevier, 2012.

[ref2] Malkin R.; Malmström B. G. The state and function of copper in biological systems. Adv. Enzymol. Relat. Areas Mol. Biol. 1970, 33, 177–244. 10.1002/9780470122785.ch4. 4318312

[ref3] Fee J. A. Copper Proteins Systems Containing the “Blue” Copper Center. In Biochemistry; Springer, 1975; pp 1–60.

[ref4] Solomon E. I.; Szilagyi R. K.; DeBeer George S.; Basumallick L. Electronic Structures of Metal Sites in Proteins and Models: Contributions to Function in Blue Copper Proteins. Chem. Rev. 2004, 104, 419–458. 10.1021/cr0206317. 14871131

[ref5] Lancaster K. M.; DeBeer George S.; Yokoyama K.; Richards J. H.; Gray H. B. Type-zero copper proteins. Nat. Chem. 2009, 1, 711 10.1038/nchem.412. 20305734 PMC2841405

[ref6] Adman E. T.; Jensen L. H. Structural features of Azurin at 2.7 Angstroms. Isr. J. Chem. 1981, 21, 8–11. 10.1002/ijch.198100003.

[ref7] Peisach J.; Blumberg W. E. Structural implications derived from the analysis of electron paramagnetic resonance spectra of natural and artificial copper proteins. Arch. Biochem. Biophys. 1974, 165, 691–708. 10.1016/0003-9861(74)90298-7. 4374138

[ref8] Williams R. J. P. Catalysis by metallo-enzymes: The entatic state. Inorg. Chim. Acta Rev. 1971, 5, 137–155. 10.1016/0073-8085(71)80016-5.

[ref9] Williams R. J. P. Energised (entatic) States of Groups and of Secondary Structures in Proteins and Metalloproteins. Eur. J. Biochem. 1995, 234, 363–381. 10.1111/j.1432-1033.1995.363_b.x. 8536678

[ref10] Ryde U.; Olsson M. H. M.; Pierloot K.; Roos B. O. The Cupric Geometry of Blue Copper Proteins is not Strained. J. Mol. Biol. 1996, 261, 586–596. 10.1006/jmbi.1996.0484. 8794878

[ref11] Stanek J.; Hoffmann A.; Herres-Pawlis S. Renaissance of the entatic state principle. Coord. Chem. Rev. 2018, 365, 103–121. 10.1016/j.ccr.2018.03.009.

[ref12] Colman P.; Freeman H.; Guss J.; Murata M.; Norris V.; Ramshaw J.; Venkatappa M. X-ray crystal structure analysis of plastocyanin at 2.7 Å resolution. Nature 1978, 272, 319–324. 10.1038/272319a0.

[ref13] Sutherland I. W.; Wilkinson J. F. Azurin – A Copper Protein Found in Bordetella. J. Gen. Microbiol. 1963, 30, 105–112. 10.1099/00221287-30-1-105. 13979392

[ref14] Gewirth A. A.; Solomon E. I. Electronic structure of plastocyanin: excited state spectral features. J. Am. Chem. Soc. 1988, 110, 3811–3819. 10.1021/ja00220a015.

[ref15] Solomon E. I.; Hare J. W.; Dooley D. M.; Dawson J. H.; Stephens P. J.; Gray H. B. Spectroscopic studies of stellacyanin, plastocyanin, and azurin. Electronic structure of the blue copper sites. J. Am. Chem. Soc. 1980, 102, 168–178. 10.1021/ja00521a029.

[ref16] Solomon E. I.; Baldwin M. J.; Lowery M. D. Electronic structures of active sites in copper proteins: contributions to reactivity. Chem. Rev. 1992, 92, 521–542. 10.1021/cr00012a003.

[ref17] Solomon E. I. Spectroscopic Methods in Bioinorganic Chemistry: Blue to Green to Red Copper Sites. Inorg. Chem. 2006, 45, 8012–8025. 10.1021/ic060450d. 16999398

[ref18] Sarangi R.; Gorelsky S. I.; Basumallick L.; Hwang H. J.; Pratt R. C.; Stack T. D. P.; Lu Y.; Hodgson K. O.; Hedman B.; Solomon E. I. Spectroscopic and Density Functional Theory Studies of the Blue–Copper Site in M121SeM and C112SeC Azurin: Cu–Se Versus Cu–S Bonding. J. Am. Chem. Soc. 2008, 130, 3866–3877. 10.1021/ja076495a. 18314977 PMC2713798

[ref19] Hadt R. G.; Sun N.; Marshall N. M.; Hodgson K. O.; Hedman B.; Lu Y.; Solomon E. I. Spectroscopic and DFT Studies of Second-Sphere Variants of the Type 1 Copper Site in Azurin: Covalent and Nonlocal Electrostatic Contributions to Reduction Potentials. J. Am. Chem. Soc. 2012, 134, 16701–16716. 10.1021/ja306438n. 22985400 PMC3506006

[ref20] Roberts J. E.; Cline J. F.; Lum V.; Gray H. B.; Freeman H.; Peisach J.; Reinhammar B.; Hoffman B. M. Comparative ENDOR study of six blue copper proteins. J. Am. Chem. Soc. 1984, 106, 5324–5330. 10.1021/ja00330a048.

[ref21] Coremans J. W. A.; Poluektov O. G.; Groenen E. J. J.; Canters G. W.; Nar H.; Messerschmidt A. A W-Band Electron Spin Echo Envelope Modulation Study of a Single Crystal of Azurin. J. Am. Chem. Soc. 1997, 119, 4726–4731. 10.1021/ja9638420.

[ref22] Bertini I.; Fernández C. O.; Karlsson B. G.; Leckner J.; Luchinat C.; Malmström B. G.; Nersissian A. M.; Pierattelli R.; Shipp E.; Valentine J. S.; et al. Structural information through NMR hyperfine shifts in blue copper proteins. J. Am. Chem. Soc. 2000, 122, 3701–3707. 10.1021/ja992674j.

[ref23] Epel B.; Slutter C. S.; Neese F.; Kroneck P. M. H.; Zumft W. G.; Pecht I.; Farver O.; Lu Y.; Goldfarb D. Electron-Mediating CuA Centers in Proteins: A Comparative High Field 1H ENDOR Study. J. Am. Chem. Soc. 2002, 124, 8152–8162. 10.1021/ja012514j. 12095361

[ref24] Fittipaldi M.; Warmerdam G. C. M.; de Waal E. C.; Canters G. W.; Cavazzini D.; Rossi G. L.; Huber M.; Groenen E. J. J. Spin-Density Distribution in the Copper Site of Azurin. ChemPhysChem 2006, 7, 1286–1293. 10.1002/cphc.200500551. 16683281

[ref25] Sottini S.; Gast P.; Blok A.; Canters G. W.; Cavazzini D.; Rossi G. L.; Groenen E. J. J. A Proton ENDOR Study of Azurin. Appl. Magn. Reson. 2010, 37, 219 10.1007/s00723-009-0048-9. 19960067 PMC2784076

[ref26] Solomon E. I.; Hedman B.; Hodgson K. O.; Dey A.; Szilagyi R. K. Ligand K-edge X-ray absorption spectroscopy: covalency of ligand–metal bonds. Coord. Chem. Rev. 2005, 249, 97–129. 10.1016/j.ccr.2004.03.020.

[ref27] Ramirez Cohen M.; Mendelman N.; Radoul M.; Wilson T. D.; Savelieff M. G.; Zimmermann H.; Kaminker I.; Feintuch A.; Lu Y.; Goldfarb D. Thiolate Spin Population of Type I Copper in Azurin Derived from 33S Hyperfine Coupling. Inorg. Chem. 2017, 56, 6163–6174. 10.1021/acs.inorgchem.7b00167. 28509562

[ref28] Lancaster K. M.; Zaballa M.-E.; Sproules S.; Sundararajan M.; DeBeer S.; Richards J. H.; Vila A. J.; Neese F.; Gray H. B. Outer-sphere contributions to the electronic structure of type zero copper proteins. J. Am. Chem. Soc. 2012, 134, 8241–8253. 10.1021/ja302190r. 22563915 PMC4794991

[ref29] Morton J. R.; Preston K. F. Atomic parameters for paramagnetic resonance data. J. Magn. Reson. 1969, 1978, 577–582. 10.1016/0022-2364(78)90284-6.

[ref30] Sugiyama A.; Sugimori K.; Shuku T.; Nakamura T.; Saito H.; Nagao H.; Kawabe H.; Nishikawa K. Electronic structure of the active site with two configurations of azurin. Int. J. Quantum Chem. 2005, 105, 588–595. 10.1002/qua.20783.

[ref31] Kang J.; Ohta T.; Hagiwara Y.; Nishikawa K.; Yamamoto T.; Nagao H.; Tateno M. Electronic and geometric structures of the blue copper site of azurin investigated by QM/MM hybrid calculations. J. Phys.: Condens. Matter 2009, 21, 064235 10.1088/0953-8984/21/6/064235. 21715937

[ref32] Remenyi C.; Reviakine R.; Kaupp M. Density Functional Study of EPR Parameters and Spin-Density Distribution of Azurin and Other Blue Copper Proteins. J. Phys. Chem. B 2007, 111, 8290–8304. 10.1021/jp071745v. 17592871

[ref33] Neese F. Theoretical Study of Ligand Superhyperfine Structure. Application to Cu(II) Complexes. J. Phys. Chem. A 2001, 105, 4290–4299. 10.1021/jp003254f.

[ref34] Neese F. Quantum Chemistry and EPR Parameters. eMagRes 2017, 6, 1–22. 10.1002/9780470034590.emrstm1505.

[ref35] Tullius T. D.; Frank P.; Hodgson K. O. Characterization of the blue copper site in oxidized azurin by extended X-ray absorption fine structure: Determination of a short Cu–S distance. Proc. Natl. Acad. Sci. U.S.A. 1978, 75, 4069–4073. 10.1073/pnas.75.9.4069. 16592557 PMC336051

[ref36] Cheung K.-C.; Strange R. W.; Hasnain S. S. 3D EXAFS refinement of the Cu site of azurin sheds light on the nature of structural change at the metal centre in an oxidation–reduction process: an integrated approach combining EXAFS and crystallography. Acta Crystallogr., Sect. D: Biol. Crystallogr. 2000, 56, 697–704. 10.1107/S0907444900003310. 10818346

[ref37] Penfield K. W.; Gewirth A. A.; Solomon E. I. Electronic structure and bonding of the blue copper site in plastocyanin. J. Am. Chem. Soc. 1985, 107, 4519–4529. 10.1021/ja00301a024.

[ref38] Larsson S.; Broo A.; Sjoelin L. Connection between structure, electronic spectrum, and electron-transfer properties of blue copper proteins. J. Phys. Chem. A. 1995, 99, 4860–4865. 10.1021/j100013a067.

[ref39] Pierloot K.; De Kerpel J. O.; Ryde U.; Roos B. O. Theoretical study of the electronic spectrum of plastocyanin. J. Am. Chem. Soc. 1997, 119, 218–226. 10.1021/ja962381f.

[ref40] Vancoillie S.; Pierloot K. Multiconfigurational g Tensor Calculations as a Probe for the Covalency of the Copper–Ligand Bonds in Copper (II) Complexes:[CuCl_4_]^2-^, [Cu(NH_3_)_4_]^2+^, and Plastocyanin. J. Phys. Chem. A 2008, 112, 4011–4019. 10.1021/jp711345n. 18386853

[ref41] Ando K. The axial methionine ligand may control the redox reorganizations in the active site of blue copper proteins. J. Chem. Phys. 2010, 133, 175101 10.1063/1.3495983. 21054068

[ref42] Ando K. Ligand-to-metal charge-transfer dynamics in a blue copper protein plastocyanin: A molecular dynamics study. J. Phys. Chem. B 2008, 112, 250–256. 10.1021/jp074822v. 18047310

[ref43] Ando K. Excited state potentials and ligand force field of a blue copper protein plastocyanin. J. Phys. Chem. B 2004, 108, 3940–3946. 10.1021/jp037412p.

[ref44] Ungar L. W.; Scherer N. F.; Voth G. A. Classical molecular dynamics simulation of the photoinduced electron transfer dynamics of plastocyanin. Biophys. J. 1997, 72, 5–17. 10.1016/S0006-3495(97)78642-9. 8994588 PMC1184292

[ref45] Deeth R. J. Comprehensive molecular mechanics model for oxidized type I copper proteins: active site structures, strain energies, and entatic bulging. Inorg. Chem. 2007, 46, 4492–4503. 10.1021/ic062399j. 17461575

[ref46] Sinnecker S.; Neese F. QM/MM calculations with DFT for taking into account protein effects on the EPR and optical spectra of metalloproteins. Plastocyanin as a case study. J. Comput. Chem. 2006, 27, 1463–1475. 10.1002/jcc.20426. 16807973

[ref47] Ryde U.; Olsson M. H. Structure, strain, and reorganization energy of blue copper models in the protein. Int. J. Quantum Chem. 2001, 81, 335–347. 10.1002/1097-461X(2001)81:5<335::AID-QUA1003>3.0.CO;2-Q.

[ref48] Olsson M. H.; Hong G.; Warshel A. Frozen density functional free energy simulations of redox proteins: computational studies of the reduction potential of plastocyanin and rusticyanin. J. Am. Chem. Soc. 2003, 125, 5025–5039. 10.1021/ja0212157. 12708852

[ref49] Riplinger C.; Neese F. An efficient and near linear scaling pair natural orbital based local coupled cluster method. J. Chem. Phys. 2013, 138, 034106 10.1063/1.4773581. 23343267

[ref50] Saitow M.; Becker U.; Riplinger C.; Valeev E. F.; Neese F. A new near-linear scaling, efficient and accurate, open-shell domain-based local pair natural orbital coupled cluster singles and doubles theory. J. Chem. Phys. 2017, 146, 164105 10.1063/1.4981521. 28456208

[ref51] Saitow M.; Neese F. Accurate spin-densities based on the domain-based local pair-natural orbital coupled-cluster theory. J. Chem. Phys. 2018, 149, 034104 10.1063/1.5027114. 30037259

[ref52] Nar H.; Messerschmidt A.; Huber R.; van de Kamp M.; Canters G. W. Crystal structure analysis of oxidized Pseudomonas aeruginosa azurin at pH 5·5 and pH 9·0: A pH-induced conformational transition involves a peptide bond flip. J. Mol. Biol. 1991, 221, 765–772. 10.1016/0022-2836(91)80173-R. 1942029

[ref53] van Gastel M.; Coremans J. W. A.; Sommerdijk H.; van Hemert M. C.; Groenen E. J. J. An ab Initio Quantum-Chemical Study of the Blue-Copper Site of Azurin. J. Am. Chem. Soc. 2002, 124, 2035–2041. 10.1021/ja0028166. 11866618

[ref54] Huang J.; Rauscher S.; Nawrocki G.; Ran T.; Feig M.; de Groot B. L.; Grubmüller H.; MacKerell A. D. CHARMM36: An Improved Force Field for Folded and Intrinsically Disordered Proteins. Biophys. J. 2017, 112, 175A–176A. 10.1016/j.bpj.2016.11.971. PMC5199616 27819658

[ref55] Melo M. C.; Bernardi R. C.; Rudack T.; Scheurer M.; Riplinger C.; Phillips J. C.; Maia J. D.; Rocha G. B.; Ribeiro J. V.; Stone J. E.; et al. NAMD goes quantum: an integrative suite for hybrid simulations. Nat. Methods 2018, 15, 351 10.1038/nmeth.4638. 29578535 PMC6095686

[ref56] Grimme S.; Ehrlich S.; Goerigk L. Effect of the damping function in dispersion corrected density functional theory. J. Comput. Chem. 2011, 32, 1456–1465. 10.1002/jcc.21759. 21370243

[ref57] Hess B. A. Relativistic electronic-structure calculations employing a two-component no-pair formalism with external-field projection operators. Phys. Rev. A 1986, 33, 3742 10.1103/PhysRevA.33.3742. 9897114

[ref58] Hess B. A. Applicability of the no-pair equation with free-particle projection operators to atomic and molecular structure calculations. Phys. Rev. A 1985, 32, 756 10.1103/PhysRevA.32.756. 9896123

[ref59] Douglas M.; Kroll N. M. Quantum electrodynamical corrections to the fine structure of helium. Ann. Phys. 1974, 82, 89–155. 10.1016/0003-4916(74)90333-9.

[ref60] Wolf A.; Reiher M.; Hess B. A. The generalized Douglas–Kroll transformation. J. Chem. Phys. 2002, 117, 9215–9226. 10.1063/1.1515314. 15267790

[ref61] van Lenthe E.; Snijders J. G.; Baerends E.-J. The zero-order regular approximation for relativistic effects: The effect of spin–orbit coupling in closed shell molecules. J. Chem. Phys. 1996, 105, 6505–6516. 10.1063/1.472460.

[ref62] van Lenthe E.; Baerends E.-J.; Snijders J. G. Relativistic total energy using regular approximations. J. Chem. Phys. 1994, 101, 9783–9792. 10.1063/1.467943.

[ref63] van Lenthe E.; Baerends E.-J.; Snijders J. G. Relativistic regular two-component Hamiltonians. J. Chem. Phys. 1993, 99, 4597–4610. 10.1063/1.466059.

[ref64] Weigend F.; Ahlrichs R. Balanced basis sets of split valence, triple zeta valence and quadruple zeta valence quality for H to Rn: Design and assessment of accuracy. Phys. Chem. Chem. Phys. 2005, 7, 3297–3305. 10.1039/b508541a. 16240044

[ref65] Pantazis D. A.; Chen X.-Y.; Landis C. R.; Neese F. All-electron scalar relativistic basis sets for third-row transition metal atoms. J. Chem. Theory Comput. 2008, 4, 908–919. 10.1021/ct800047t. 26621232

[ref66] Weigend F. Accurate Coulomb-fitting basis sets for H to Rn. Phys. Chem. Chem. Phys. 2006, 8, 1057–1065. 10.1039/b515623h. 16633586

[ref67] Neese F.; Wennmohs F.; Hansen A.; Becker U. Efficient, approximate and parallel Hartree–Fock and hybrid DFT calculations. A ‘chain-of-spheres’ algorithm for the Hartree–Fock exchange. Chem. Phys. 2009, 356, 98–109. 10.1016/j.chemphys.2008.10.036.

[ref68] Tao J.; Perdew J. P.; Staroverov V. N.; Scuseria G. E. Climbing the density functional ladder: Nonempirical meta–generalized gradient approximation designed for molecules and solids. Phys. Rev. Lett. 2003, 91, 146401 10.1103/PhysRevLett.91.146401. 14611541

[ref69] Stoychev G. L.; Auer A. A.; Neese F. Automatic Generation of Auxiliary Basis Sets. J. Chem. Theory Comput. 2017, 13, 554–562. 10.1021/acs.jctc.6b01041. 28005364

[ref70] Visscher L.; Dyall K. G. Dirac–Fock atomic electronic structure calculations using different nuclear charge distributions. At. Data Nucl. Data Tables 1997, 67, 207–224. 10.1006/adnd.1997.0751.

[ref71] Sandhoefer B.; Neese F. One-electron contributions to the g-tensor for second-order Douglas–Kroll–Hess theory. J. Chem. Phys. 2012, 137, 094102 10.1063/1.4747454. 22957550

[ref72] Neese F. Prediction and interpretation of the 57Fe isomer shift in Mössbauer spectra by density functional theory. Inorg. Chim. Acta 2002, 337, 181–192. 10.1016/S0020-1693(02)01031-9.

[ref73] Sparta M.; Retegan M.; Pinski P.; Riplinger C.; Becker U.; Neese F. Multilevel Approaches within the Local Pair Natural Orbital Framework. J. Chem. Theory Comput. 2017, 13, 3198–3207. 10.1021/acs.jctc.7b00260. 28590754

[ref74] Stoll S.; Schweiger A. EasySpin, a comprehensive software package for spectral simulation and analysis in EPR. J. Magn. Reson. 2006, 178, 42–55. 10.1016/j.jmr.2005.08.013. 16188474

[ref75] Dodd F. E.; Abraham Z. H. L.; Eady R. R.; Hasnain S. S. Structures of oxidized and reduced azurin II from *Alcaligenes xylosoxidans* at 1.75 A resolution. Acta Crystallogr., Sect. D: Biol. Crystallogr. 2000, 56, 690–696. 10.1107/S0907444900003309. 10818345

[ref76] Chen Z.-W.; Barber M. J.; McIntire W. S.; Mathews F. S. Crystallographic Study of Azurin from *Pseudomonas putida*. Acta Crystallogr., Sect. D: Biol. Crystallogr. 1998, 54, 253–268. 10.1107/S0907444997011505. 9761890

[ref77] Paraskevopoulos K.; Sundararajan M.; Surendran R.; Hough M. A.; Eady R. R.; Hillier I. H.; Hasnain S. S. Active site structures and the redox properties of blue copper proteins: atomic resolution structure of azurin II and electronic structure calculations of azurin, plastocyanin and stellacyanin. Dalton Trans. 2006, 126, 3067–3076. 10.1039/b513942b. 16786065

[ref78] Baker E. N. Structure of azurin from Alcaligenes denitrificans refinement at 1·8 Å resolution and comparison of the two crystallographically independent molecules. J. Mol. Biol. 1988, 203, 1071–1095. 10.1016/0022-2836(88)90129-5. 3210236

[ref79] Staroverov V. N.; Scuseria G. E.; Tao J.; Perdew J. P. Comparative assessment of a new nonempirical density functional: Molecules and hydrogen-bonded complexes. J. Chem. Phys. 2003, 119, 12129–12137. 10.1063/1.1626543.

[ref80] Stephens P.; Devlin F.; Chabalowski C.; Frisch M. J. Ab initio calculation of vibrational absorption and circular dichroism spectra using density functional force fields. J. Phys. Chem. B. 1994, 98, 11623–11627. 10.1021/j100096a001.

[ref81] Caldeweyher E.; Bannwarth C.; Grimme S. Extension of the D3 dispersion coefficient model. J. Chem. Phys. 2017, 147, 034112 10.1063/1.4993215. 28734285

[ref82] Gómez-Piñeiro R. J.; Pantazis D. A.; Orio M. Comparison of Density Functional and Correlated Wave Function Methods for the Prediction of Cu(II) Hyperfine Coupling Constants. ChemPhysChem 2020, 21, 2667–2679. 10.1002/cphc.202000649. 33201578 PMC7756273

[ref83] Coremans J. W. A.; van Gastel M.; Poluektov O. G.; Groenen E. J. J.; den Blaauwen T.; van Pouderoyen G.; Canters G. W.; Nar H.; Hammann C.; Messerschmidt A. An ENDOR and ESEEM study of the blue copper protein azurin. Chem. Phys. Lett. 1995, 235, 202–210. 10.1016/0009-2614(95)00114-J.

[ref84] Werst M. M.; Davoust C. E.; Hoffman B. M. Ligand spin densities in blue copper proteins by q-band proton and nitrogen-14 ENDOR spectroscopy. J. Am. Chem. Soc. 1991, 113, 1533–1538. 10.1021/ja00005a011.

[ref85] Neese F.; Hedman B.; Hodgson K. O.; Solomon E. I. Relationship between the Dipole Strength of Ligand Pre-Edge Transitions and Metal– Ligand Covalency. Inorg. Chem. 1999, 38, 4854–4860. 10.1021/ic990461p. 11671216

[ref86] Ray K.; DeBeer George S.; Solomon E. I.; Wieghardt K.; Neese F. Description of the ground-state covalencies of the bis (Dithiolato) transition-metal complexes from X-ray absorption spectroscopy and time-dependent density-functional calculations. Chem. – Eur. J. 2007, 13, 2783–2797. 10.1002/chem.200601425. 17290468

[ref87] DeBeer George S.; Petrenko T.; Neese F. Time-dependent density functional calculations of ligand K-edge X-ray absorption spectra. Inorg. Chim. Acta 2008, 361, 965–972. 10.1016/j.ica.2007.05.046.

[ref88] Neese F. Metal and ligand hyperfine couplings in transition metal complexes: The effect of spin–orbit coupling as studied by coupled perturbed Kohn–Sham theory. J. Chem. Phys. 2003, 118, 3939–3948. 10.1063/1.1540619.

