# Supplementary material for: Converged Structural and Spectroscopic Properties for Refined QM/MM Models of Azurin
Source: Inorg Chem. 2021 May 3;60(10):7399–412. doi: 10.1021/acs.inorgchem.1c00640 (PMC8154437; doi:10.1021/acs.inorgchem.1c00640)
Supplement: Supplementary file 1 — ic1c00640_si_001.pdf [file ic1c00640_si_001.pdf]

SUPPORTING INFORMATION

for

Converged Structural and Spectroscopic Properties for Refined QM/MM Models of Azurin

Christine E. Schulz, Maurice van Gastel, Dimitrios A. Pantazis, Frank Neese

Table S1. Distances (in Å) between Cu and surrounding atoms in the reduced state of azurin as obtained from previous studies reported in the literature.

	Cu–S_{Cys112}	Cu–N_{His117}	Cu–N_{His46}	Cu–S_{Met121}	Cu–O_{Gly45}
Ref. [1] (high type)	2.24	1.95	2.04	3.20	2.87
Ref. [1] (low type)	2.25	1.95	2.06	3.17	2.84
Ref. [2] MD–MM	2.25	1.95	2.06	3.35	2.96
Ref. [2] Model I (electrostatic embedding)	2.20	2.00	2.03	3.49	3.01
Ref. [2] Model II (only VdW embedding)	2.24	2.10	1.93	3.50	2.81
Ref. [3] Gas phase[a]	2.14	2.00	2.00	3.36	2.94
Ref. [3] QM/MM(EE)[a]	2.17	2.01	1.99	3.53	2.55
Ref. [3] QM/MM(ME)[a]	2.17	2.03	2.01	3.41	2.49
Ref. [4] QM/MM[b]	2.18	1.98	1.99	3.18	2.84

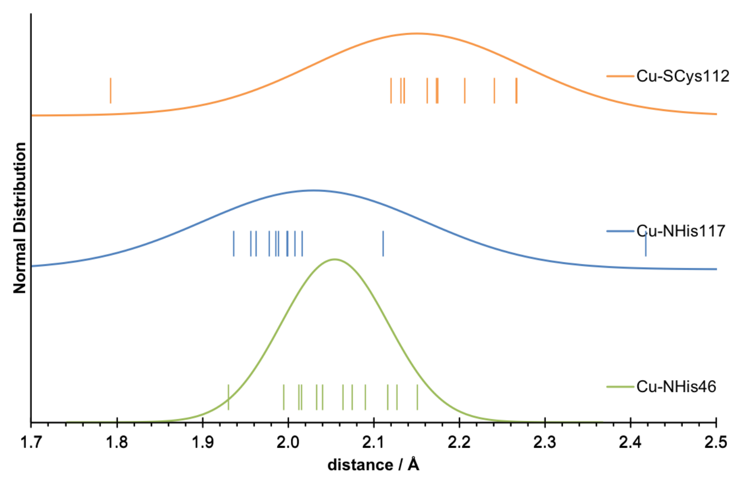

Figure S1. Graphical analysis of the crystal structure averages for the equatorial ligand distances of the copper active site.

Selection of DFT functional

The main criterion is the critical Cu-S_{Cys} distance. In general, most functionals perform similarly (Table S2). No sharp grouping in terms of pure GGA and hybrid functionals is visible. B3LYP and M06 yield the longest Cu-S_{Cys} distance, B2PLYP yields the shortest Cu-S_{Cys} distance. Among the pure and hybrid functionals, BP86, TPSS, and TPSSh yield the shortest Cu-S_{Cys} distances and therefore become valuable candidates. The Cu-His distances are similar for all functionals. The distances to the weaker ligands vary, as expected. The Cu-S_{Met} distance can be grouped in to 3.05 Å for PBE, TPSS, TPSSh or 3.11 Å for M06L, B3LYP, PBE0, M06, and B2PLYP. Only BP86 shows a shorter distance of 2.98 Å. The Cu-O_{Gly} distance is either in the range of 2.87 Å for M06L, B3LYP, M06, or B2PLYP or in the range of 2.91 Å for the rest.

Table S2. Distances (in Å) between Cu and surrounding atoms in the reduced state of azurin as obtained from QM/MM optimizations for model B with different functionals.

	Cu-S_{Cys112}	Cu-N_{His117}	Cu-N_{His46}	Cu-S_{Met121}	Cu-O_{Gly45}
BP86	2.16	1.94	1.93	2.98	2.92
PBE	2.17	1.95	1.94	3.05	2.91
TPSS	2.16	1.94	1.94	3.02	2.91
M06L	2.17	1.96	1.96	3.12	2.86
B3LYP	2.18	1.95	1.95	3.10	2.87
TPSSh	2.16	1.94	1.94	3.05	2.91
PBE0	2.17	1.94	1.93	3.10	2.91
M06	2.18	1.95	1.95	3.10	2.87
B2PLYP	2.15	1.93	1.93	3.11	2.87

Table S3. Behavior of active site angles and dihedrals calculated with TPSSh-D3/DKH-def2-TZVP(-f) with increasing model size. All values in degrees. Labels as given in Figure S2.

	C_β-SCys-Cu	C_α-C_β-SCys	H_{46} N_ε-N_δ-Cu	H_{117} N_ε-N_δ-Cu	H_{46} N_ε-N_δ-Cu S	H_{117} N_ε-N_δ-Cu S	H_{117} N_ε-N_δ H_{46} N_δ-N_ε
A	117.28	112.53	160.34	159.33	143.38	133.02	-32.38
B	113.75	113.11	159.83	160.46	155.60	125.39	-30.90
C	114.65	112.97	159.02	161.24	150.22	125.54	-32.18
D	112.78	112.14	159.91	157.84	145.52	127.20	-35.41
E	111.26	112.53	159.35	158.52	147.53	125.06	-35.61
F	111.74	112.55	158.90	158.67	148.51	125.93	-35.10
G	112.80	112.28	158.83	158.43	147.38	122.34	-36.95

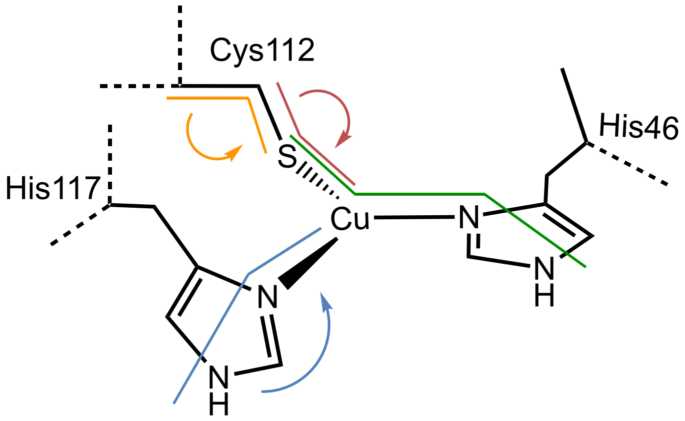

Figure S2. Schematic representation of the Cu active site angles relevant in Table S3. Cysteine angles (C_α-C_β-S_{Cys}, C_β-S_{Cys}-Cu) are shown in orange and red. The angles between the histidine imidazole ring and the copper (N_ε-N_δ-Cu) are shown for His_{117} in blue. The dihedrals between the histidine imidazole ring, the copper and SCys (N_ε-N_δ-Cu-S) are shown for His_{46} in green.

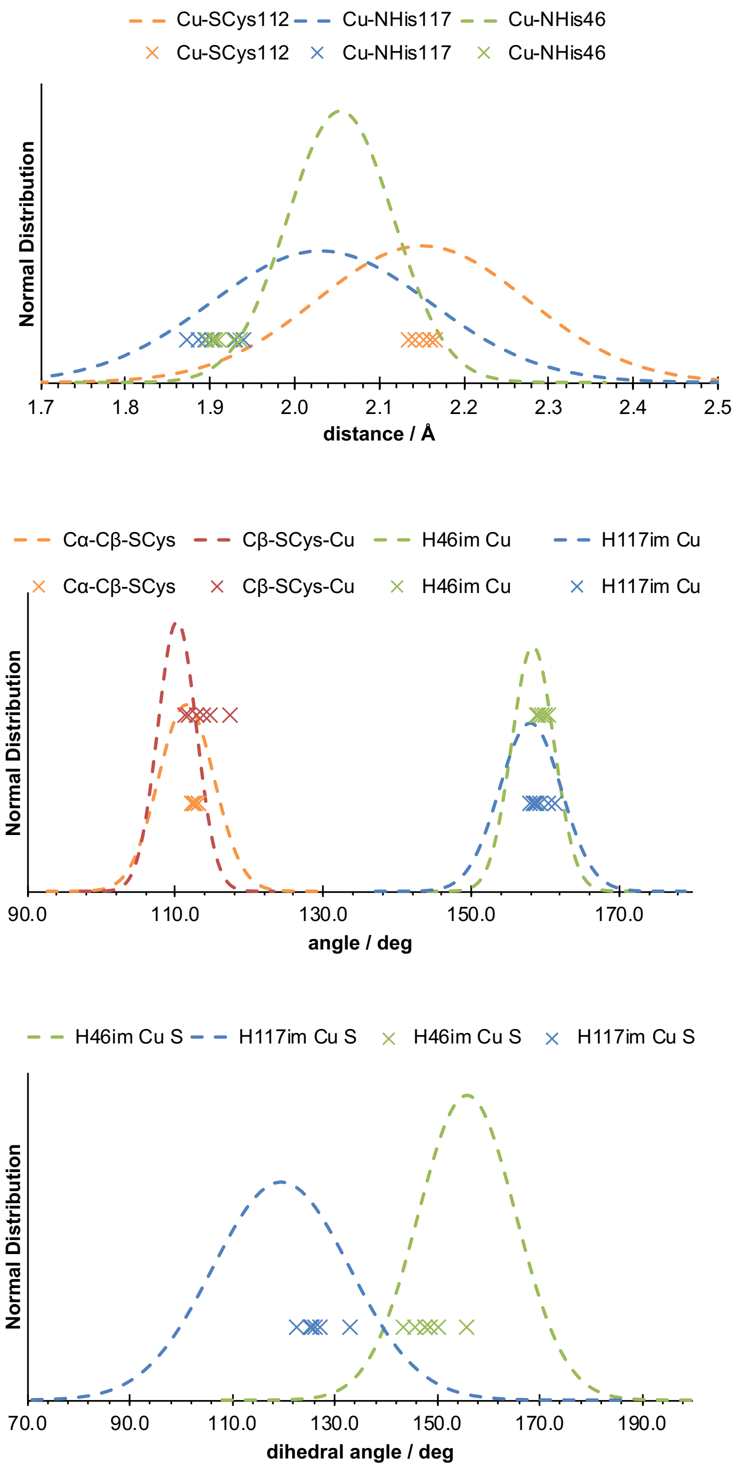

Figure S3. Comparison of distances (top), angles (middle), and dihedral angles from QM/MM optimized structures with the crystal structure normal distribution. The same color scheme as used in Figure S2.

Table S4. Cu-ligand distances from QM optimized models calculated with TPSSh-D3/DKH-def2-TZVP(-f). All values in Å.

	Cu-S_{Cys112}	Cu-N_{His117}	Cu-N_{His46}	Cu-S_{Met121}	Cu-O_{Gly45}
A	2.13	1.93	1.95		2.69
B	2.14	1.94	1.93	2.89	3.01
C	2.12	1.94	1.93	2.89	3.02
D	2.13	1.91	1.91	3.05	2.90
E	2.13	1.91	1.90	3.01	2.93
F	2.12	1.89	1.90	2.95	3.02
G	2.12	1.87	1.90	3.03	2.93

Table S5. Model A to C DLPNO-CCSD hyperfine coupling constants (MHz). Tight thresholds were applied in fragment 1 as defined in the text, thresholds applied in fragment 2 were varied accordingly.

	S_{Cys112}	N_{His117}	N_{His46}	H_A	H_B
A(loose)	19.6	23.8	21.3	21.6	9.0
A(normal)	20.1	23.9	21.6	25.3	9.7
A(tight)	20.5	23.9	21.7	27.9	11.5

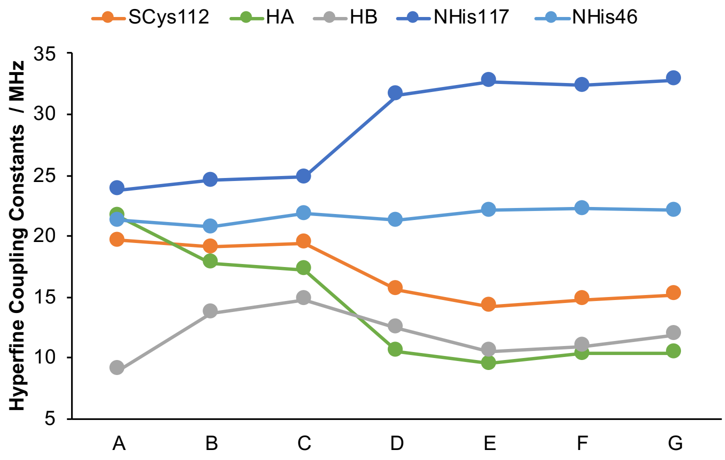

Figure S4. Graphical representation of the DLPNO-CCSD HFCs with the two-component scheme.

Table S6. Principal values of the DLPNO-CCSD HFCs (MHz) for models A to G. Tight thresholds were applied in fragment 1 as defined in the main text, LoosePNO thresholds were applied in fragment 2.

Cu	A_{xx}	A_{yy}	A_{zz}
A	4.314	-25.410	-660.756
B	-20.741	20.907	-669.866
C	10.807	-22.037	-672.535
D	-2.967	36.103	-703.380
E	1.709	27.550	-728.660
F	0.090	18.545	-724.819
G	-1.641	20.449	-717.901

SCys112	A_{xx}	A_{yy}	A_{zz}
A	-12.933	-15.119	86.897
B	-11.389	-13.615	82.267
C	-11.333	-13.521	83.113
D	-8.345	-10.129	65.256
E	-6.991	-8.641	58.261
F	-7.425	-9.121	60.847
G	-7.285	-9.072	61.975

N_{δ}His117	A_{xx}	A_{yy}	A_{zz}
A	21.321	21.920	28.174
B	22.046	22.727	28.920
C	22.325	23.006	29.258
D	28.262	29.089	37.433
E	29.175	30.022	38.988
F	28.818	29.661	38.616

G	29.183	30.056	39.220

N_{δ}His46	A_{xx}	A_{yy}	A_{zz}
A	18.831	19.509	25.424
B	18.510	19.042	24.710
C	19.464	20.017	25.940
D	18.829	19.389	25.720
E	19.574	20.156	26.652
F	19.708	20.288	26.739
G	19.553	20.138	26.556

H_A	A_{xx}	A_{yy}	A_{zz}
A	19.458	19.784	25.649
B	15.461	16.257	21.714
C	14.846	15.789	21.209
D	8.246	9.270	14.171
E	8.246	9.270	14.171
F	7.234	8.219	13.099
G	8.009	9.001	13.936

H_B	A_{xx}	A_{yy}	A_{zz}
A	6.245	7.597	13.193
B	10.960	12.162	17.863
C	12.184	13.247	18.978
D	12.184	13.247	18.978
E	10.073	10.961	16.447
F	8.094	9.010	14.449
G	8.545	9.463	14.942

Table S7. Mulliken and Löwdin spin populations calculated using DLPNO-CCSD on model C.

	Mulliken	Löwdin
Cu	0.5242	0.5822
Cys112 S	0.3714	0.3143
Cys112 H_A	0.0101	0.0057
Cys112 H_B	0.0092	0.0047
His46 $C_\varepsilon H$	0.0007	-0.0001
His46 $N_\varepsilon H$	0.0006	0.0004
His46 N_δ	0.0501	0.0346
His46 N_ε	-0.0002	-0.0001
His117 $C_\varepsilon H$	0.0008	0.0001
His117 $N_\varepsilon H$	0.0001	0.0003
His117 N_δ	0.0532	0.0378
His117 N_ε	-0.0003	0.0000

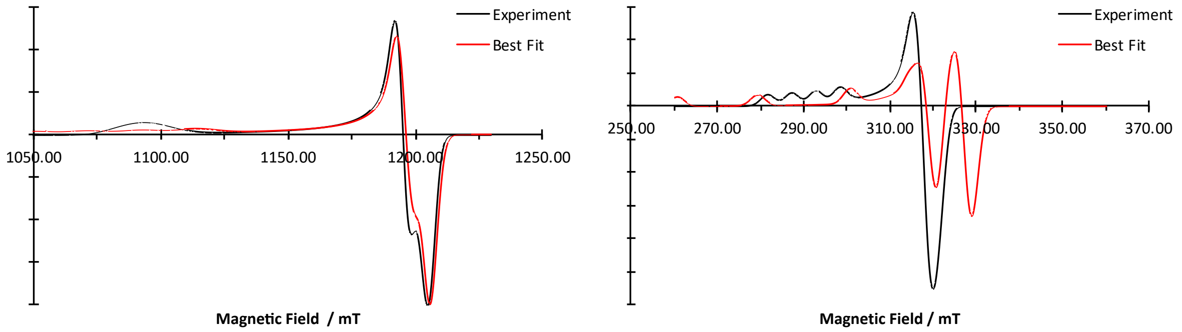

Figure S5. Simulations of the Q-band and W-band EPR spectrum using the experimental Cu HFCs and *g*-tensor (black lines) *vs* the experimental *g*-tensor[5] and DLPNO-CCSD Cu HFC.

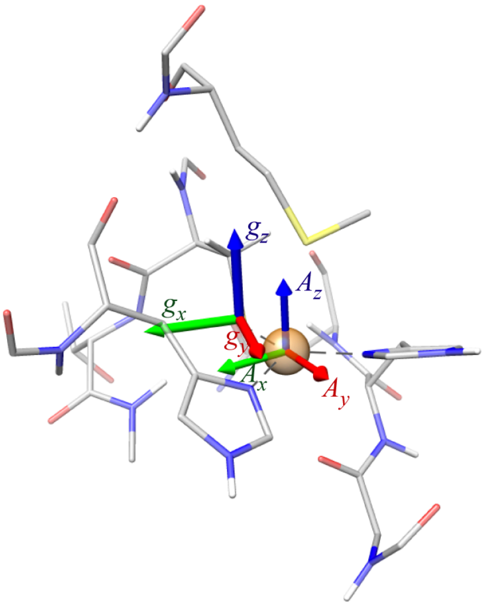

Figure S6. Orientation of the copper hyperfine tensor (shown centered on Cu) with respect to the experimentally determined *g* tensor.

Rotation matrices used in spectral simulations:

Histidine Nδ(His46) from calculations vs experiment.

	A_x	A_y	A_z		A_x	A_y	A_z
	19.6	20.2	25.8		19.1	18	17.2
	a_x	a_y	a_z		a_x	a_y	a_z
a	-0.369	0.702	0.608	a	0.607	0.399	-0.687
b	0.616	0.675	-0.406	b	-0.414	-0.578	-0.704
c	-0.696	0.225	-0.682	c	-0.678	0.712	-0.185

Histidine Nδ(His117) from calculations vs experiment.

	A_x	A_y	A_z		A_x	A_y	A_z
	22.7	23.4	29.4		27.8	24.0	23.6
	a_x	a_y	a_z		a_x	a_y	a_z
a	0.051	-0.594	0.803	a	-0.784	-0.009	0.621
b	0.873	-0.365	-0.325	b	0.320	0.851	0.418
c	0.486	0.717	0.499	c	-0.532	0.526	-0.664

Cysteine beta proton HβA from calculations vs Hβ2 from experiment.

	A_x	A_y	A_z		A_x	A_y	A_z
	18.7	19.8	25.0		14.4	19.1	23.0
	a_x	a_y	a_z		a_x	a_y	a_z
g_x	-0.680	-0.698	-0.225	g_x	-0.805	-0.504	-0.314
g_y	-0.489	0.660	-0.570	g_y	-0.316	0.811	-0.492
g_z	0.546	-0.277	-0.791	g_z	0.503	-0.297	-0.812

Cysteine beta proton HβB from calculations vs Hβ1 from experiment.

	A_x	A_y	A_z		A_x	A_y	A_z
	15.9	17.1	22.6		20.4	21.3	26.2
	a_x	a_y	a_z		a_x	a_y	a_z
g_x	-0.920	-0.261	0.292	g_x	0.955	-0.272	-0.114
g_y	-0.354	0.873	-0.334	g_y	0.285	0.752	0.594
g_z	-0.169	-0.411	-0.896	g_z	-0.080	-0.600	0.796

Table S8. Calculated Quadrupole splittings from DLPNO-CCSD.

	Q_x	Q_y	Q_z
S Cys_{112}	0.0	-3.5	-4.3
N His_{117}	0.0	-0.8	-1.1
N His_{46}	0.0	6.6	-8.5

Table S9. Mulliken spin populations of Cu and S_{Cys112} as reported in previous studies.

	Cu	S_{Cys112}
B3LYP[6]	0.29	0.62
B3LYP/DKH[6]	0.33	0.58
high type[1a]	0.51	0.41
low type[1a]	0.52	0.41
Model I[2b]	0.39	0.51
Model II[2c]	0.34	0.57
Loewdin		
QM/MM[4]	0.508	0.363

a QM single points on MM geometries using UB3LYP/6-31G*.
b QM/MM cluster model electrostatic embedding, B3LYP/6-31G*, TZVP on Cu, 65 atoms.
c QM/MM cluster model only vdW embedding, B3LYP/6-31G*, TZVP on Cu, 65 atoms

Simulation of 2D-EDNMR spectra

To overcome the resolution issues in the 1D-EDNMR, 2D-EDNMR spectra were recorded.[27] Therefore, a final comparison with experiment involves the 2D-EDNMR spectra,[27] as shown in Figure S6. The experimental spectrum is shown in the bottom row, while the simulation using the calculated parameters is shown in the top row. These spectra are much more complex than the 1D-EDNMR, as they involve corrleations between different transitions of the same nucleus. However, from experiments with unlabeled azurin[27, 84] it is known the ^{14}N signals appear on the axes because the nuclear tansitons of one of the electron spin manifolds has very low frequency. Therefore, all cross peaks observed in the labeled azurin are originating from the ^{33}S. Looking at these cross peaks in more detail, in the 3048 mT spectrum, referring to the *g* parallel position, a good agreement is observed. In the (++) quadrant, cross peaks are observed at (19,38) MHz and (19,56) MHz, both in experiment and simulation. In the (− +) quadrant, good agreement is observed for the cross peak at (23,35) MHz. Another peak appears at (23,60) MHz in the experiment and (23,52) MHz for the simulation. These differences could originate from differences in the calculated orientations of the hyperfine tensor with respect to the *g* tensor, or in differences in the quadrupole tensor. While for I=1/2 or I=1 featuring models, a relatively clear assignment of the various transitions is possible,[84-85] this is not the case for ^{33}S for which I=3/2, where the hyperfine anisotropy is large and the quadruple interaction is large as well. In the 3322 mT spectrum, referring to the *g* perpendicular position, an even more complicated situation is presented. Here multiple orientations are contributing to the final spectrum, which means that deviations in the orientations will lead to more differences in the simulated spectrum using the calculated parameters. The lack of sample spectra of intermediate complexity prevents a more detailed analysis at this point.

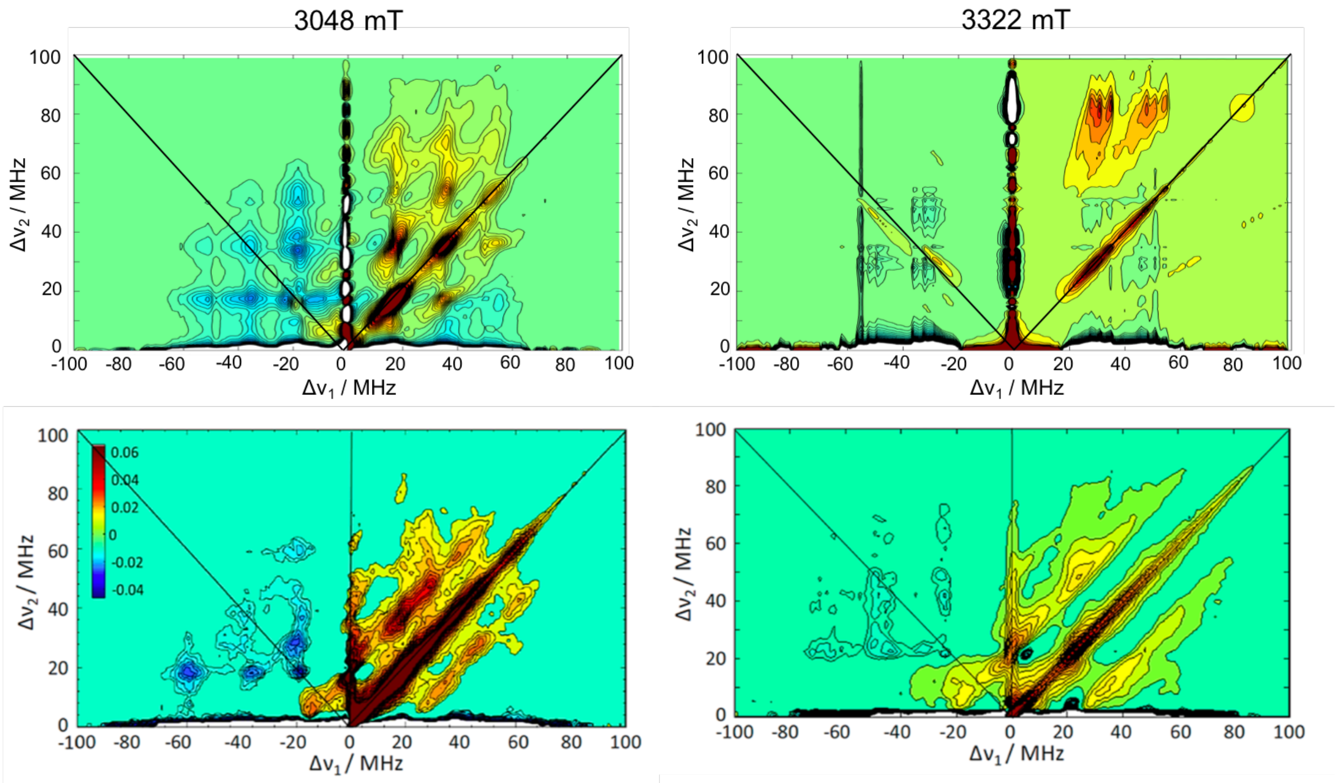

Figure S7. Comparison of the simulated 2D-EDNMR spectra obtained from DLPNO-CCSD calculations (top) with the experiment[27] (bottom) at 3048 mT (left) and 3322 mT (right). Microwave frequency: $\nu = 94.9$ GHz.

A note on the performance of DFT

Here we comment on the comparison of the results presented in the main text with those that can be obtained by density functional theory. The advantage of using DFT is that the complete series of QM regions can be considered without significant computational cost, whereas the major disadvantage is the strong variability of results depending on the nature of the functional. The functional dependence was tested using a small range of functionals (Table S10). In agreement with previous studies,[6] the expected correlations with the amount of Hartree-Fock exchange were observed, but no systematic improvement of all ligand HFCs was possible through variation of the fractional HF exchange. The TPSSh functional is used here, since it has been shown to be broadly successful in computing magnetic and spectroscopic properties.[7]

Table S10. Hyperfine coupling constants A_{iso} (MHz) calculated for model B with different functionals.

	H_A	H_B	S_{Cys}	N_{His117}	N_{His46}
TPSS	35.1	30.3	17.8	26.8	21.3
TPSSh	31.2	26.9	19.3	28.2	22.0
TPSS0	23.0	19.8	19.3	29.5	23.3
BP86	36.1	31.5	8.4	27.4	22.1
B3LYP	28.4	24.8	15.2	30.5	23.8

HFCs calculated using TPSSh for all QM/MM models are listed in Table S11 (see also Figure S8). The results show that the convergence with system size is not monotonic and that small discontinuities appear. As shown in our initial test using various DFT functionals (Table S10) the histidine nitrogen HFCs are rather stable (within 4 MHz) while the β-hydrogens show larger deviations (within 13 MHz). These variations in the β-hydrogens are in agreement with previous computational studies, which showed even larger variations depending on the model and method used.[4,6,8] This can be a serious problem because as shown in the main text,

deviations of less than 1 MHz can yield disagreement with the experimental spectrum. Therefore, the fluctuation among the DFT functionals and the intrinsically limited accuracy does not allow for a comparison and evaluation with experiment at the level of detail that can be achieved with the wave function based methods reported in the main text.

Table S11. TPSSh hyperfine coupling constants (MHz) calculated for the QM/MM optimized models.

	H_A	H_B	SCys112	N_δHis117	N_δHis46
**A**	29.4	26.3	19.2	29.9	22.7
**B**	31.2	26.9	19.3	28.2	22.0
**C**	27.6	24.8	18.3	29.0	25.4
**D**	19.5	23.6	15.5	35.3	24.5
**E**	20.8	23.2	15.7	34.7	24.8
**F**	22.6	24.5	16.5	34.1	24.6
**G**	21.7	24.8	17.0	33.8	24.2

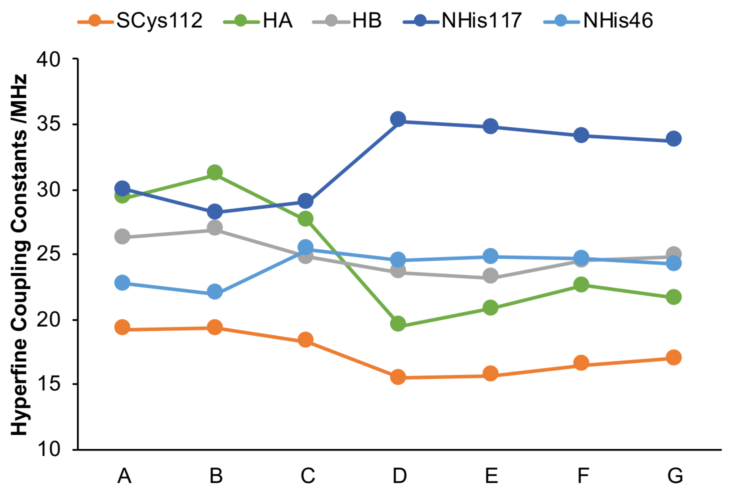

Figure S8. Hyperfine coupling constants (in MHz) of different sized models, calculated with TPSSh-D3.

Table S12. Löwdin and Mulliken spin populations of Cu and S_{Cys112} centers obtained by the different QM/MM optimized models calculated with TPSSh-D3.

	Cu		S_{Cys112}	
	Löwdin	Mulliken	Löwdin	Mulliken
A	0.51	0.45	0.33	0.38
B	0.50	0.44	0.35	0.42
C	0.52	0.47	0.31	0.36
D	0.55	0.49	0.26	0.30
E	0.54	0.49	0.27	0.30
F	0.53	0.48	0.28	0.32
G	0.53	0.47	0.28	0.32

REFERENCES

(1) Sugiyama, A.; Sugimori, K.; Shuku, T.; Nakamura, T.; Saito, H.; Nagao, H.; Kawabe, H.; Nishikawa, K. Electronic structure of the active site with two configurations of azurin. *Int. J. Quantum Chem.* **2005**, *105*, 588-595.

(2) Jiyoung, K.; Takehiro, O.; Yohsuke, H.; Keigo, N.; Tetsunori, Y.; Hidemi, N.; Masaru, T. Electronic and geometric structures of the blue copper site of azurin investigated by QM/MM hybrid calculations. *J. Phys.: Condens. Matter* **2009**, *21*, 064235.

(3) Paraskevopoulos, K.; Sundararajan, M.; Surendran, R.; Hough, M. A.; Eady, R. R.; Hillier, I. H.; Hasnain, S. S. Active site structures and the redox properties of blue copper proteins: atomic resolution structure of azurin II and electronic structure calculations of azurin, plastocyanin and stellacyanin. *Dalton Trans.* **2006**, 3067-3076.

(4) Lancaster, K. M.; Zaballa, M.-E.; Sproules, S.; Sundararajan, M.; DeBeer, S.; Richards, J. H.; Vila, A. J.; Neese, F.; Gray, H. B. Outer-sphere contributions to the electronic structure of type zero copper proteins. *J. Am. Chem. Soc.* **2012**, *134*, 8241-8253.

(5) Coremans, J. W. A.; van Gastel, M.; Poluektov, O. G.; Groenen, E. J. J.; den Blaauwen, T.; van Pouderoyen, G.; Canters, G. W.; Nar, H.; Hammann, C.; Messerschmidt, A. An ENDOR and ESEEM study of the blue copper protein azurin. *Chem. Phys. Lett.* **1995**, *235*, 202-210.

(6) Remenyi, C.; Reviakine, R.; Kaupp, M. Density Functional Study of EPR Parameters and Spin-Density Distribution of Azurin and Other Blue Copper Proteins. *J. Phys. Chem. B* **2007**, *111*, 8290-8304.

(7) Kossmann, S.; Kirchner, B.; Neese, F. Performance of modern density functional theory for the prediction of hyperfine structure: meta-GGA and double hybrid functionals. *Mol. Phys.* **2007**, *105*, 2049-2071.

(8) Moon, S.; Patchkovskii, S.; Salahub, D. R. QM/MM calculations of EPR hyperfine coupling constants in blue copper proteins. *J. Mol. Struct. (THEOCHEM)* **2003**, *632*, 287-295.
